# *circTGFBR2(3-6)* acts as an assembly platform for RNA-binding protein IGF2BP3 and *TGFBR1* mRNA to enhance breast cancer cell plasticity

**DOI:** 10.1038/s41418-025-01597-2

**Published:** 2025-10-27

**Authors:** Qian Wang, Rayman T. N. Tjokrodirijo, Hailiang Mei, Peter A. van Veelen, Peter ten Dijke, Chuannan Fan

**Affiliations:** 1 Oncode Institute and Department of Cell and Chemical Biology, Leiden University Medical Center, Leiden, The Netherlands; 2 Center for Proteomics and Metabolomics, Leiden University Medical Center, Leiden, The Netherlands; 3 Sequencing Analysis Support Core, Department of Biomedical Data Sciences, Leiden University Medical Center, Leiden, The Netherlands

**Keywords:** Metastasis, Cell biology, Molecular biology

## Abstract

Transforming growth factor (TGF)-β signaling is a key driver to induce epithelial-to-mesenchymal transition (EMT), a process that enhances cancer cell plasticity and metastatic potential. However, the role of circular RNAs (circRNAs) in TGF-β signaling remains largely unexplored. Here, we identify *circTGFBR2(3-6)*, a circRNA derived from *TGF-β receptor 2* (*TGFBR2*) pre-mRNA, as a critical enhancer of TGF-β/SMAD signaling in breast cancer cells. Depletion of *circTGFBR2(3-6)* inhibits TGF-β-induced EMT, cell migration, and in vivo extravasation of breast cancer cells. Mechanistically, *circTGFBR2(3-6)* acts as a scaffold that facilitates the interaction between the RNA-binding protein insulin-like growth factor 2 mRNA binding protein 3 (IGF2BP3) and *TGF-β receptor 1* (*TGFBR1*) mRNA in an *N*^6^-methyladenosine (m^6^A)-dependent manner, and thereby stabilizes *TGFBR1* mRNA and promotes its expression. Furthermore, *IGF2BP3* knockdown reduces *circTGFBR2(3-6)*-mediated enhancement of TGF-β/SMAD signaling, as well as TGF-β-induced EMT and cell migration. Our findings identify *circTGFBR2(3-6)* as a novel potentiator of TGF-β/SMAD signaling at the receptor level and highlight IGF2BP3 as a critical m^6^A reader that mediates *circTGFBR2(3-6)*-driven breast cancer cell plasticity.

## Introduction

Epithelial-to-mesenchymal transition (EMT) endows epithelial cancer cells with the plasticity required to transition into a mesenchymal state [1]. Mesenchymal cancer cells gain enhanced migratory and invasive abilities, allowing them to detach from primary tumors and form metastases in secondary organs [2, 3]. EMT is characterized by the downregulation of epithelial markers, such as E-cadherin, and the upregulation of mesenchymal markers, including N-cadherin, Fibronectin, and Vimentin [3, 4]. Most cancer cells undergo a hybrid/partial EMT, which confers increased aggressiveness, stem cell-like properties, and resistance to chemotherapy [5–8].

Transforming growth factor (TGF)-β signaling plays a pivotal role in inducing EMT and driving cancer progression [9, 10]. TGF-β cytokine binds to its receptor 1 (TGFBR1) and receptor 2 (TGFBR2), enabling TGFBR1 to recruit and phosphorylate SMAD2 and SMAD3 (SMAD2/3) at two carboxy-terminal serine residues [11, 12]. The complexes formed by the activated SMAD2/3 and SMAD4 translocate into the nucleus, whereby they induce the transcription of target genes, such as *SERPINE1* (encoding plasminogen activator inhibitor 1, PAI-1), *CCN2* (encoding connective tissue growth factor, CTGF), and *SNAI1* (encoding SNAIL transcriptional repressor 1, SNAI1) to regulate cellular processes like EMT [12, 13].

*N*^6^-methyladenosine (m^6^A), the most abundant internal modification on eukaryotic mRNAs, plays a crucial role in post-transcriptional gene regulation [14, 15]. m^6^A is installed by methyltransferases (referred to as “writers”), such as methyltransferase-like protein 3 (METTL3) and METTL14 [16], and removed by demethylases (referred to as “erasers”), including Fat mass and obesity-associated protein (FTO) [17] and AlkB Homolog 5 (ALKBH5) [18]. m^6^A-modified mRNAs are recognized by RNA-binding “reader” proteins, including insulin-like growth factor 2 mRNA-binding protein (IGF2BP) family members [19], to influence various aspects of mRNA cellular fate, including alternative splicing, nuclear export, stability, and translation [14, 20]. Dysregulated expression of m^6^A modifiers and aberrant global m^6^A levels are associated with cancer progression and clinical outcome [15, 21]. Notably, aberrant upregulation of METTL3 and METTL14 in cancer cells increases m^6^A accumulation on key EMT-inducing mRNAs, thereby enhancing their stability and promoting EMT [22–24].

Circular RNAs (circRNAs) are a class of covalently closed, single-stranded RNA molecules formed through back-splicing of precursor mRNA (pre-mRNA) transcripts [25, 26]. Their circular structure confers increased resistance to exonuclease-mediated degradation (e.g., by RNase R), resulting in enhanced stability than their cognate linear mRNAs [25, 26]. Although initially regarded as splicing anomalies, circRNAs are emerging as functional RNA molecules with regulatory roles distinct from their parental mRNAs. circRNAs can function as scaffolds or decoys to influence macromolecular interactions, such as RNA-protein and protein-protein interactions [27–29]. Additionally, circRNAs can sponge microRNAs (miRNAs) to prevent them from binding to their target mRNAs [30, 31]. Some circRNAs are able to be translated into functional peptides [32, 33]. Increasing evidence suggests that circRNA dysregulation contributes to cancer development and progression [34, 35].

In this study, we investigated whether circRNAs derived from the pre-mRNAs of *TGFBR1* and *TGFBR2* affect TGF-β/SMAD signaling. We identified *circTGFBR2(3-6)* as a potent enhancer of TGF-β/SMAD signaling. *circTGFBR2(3-6)* functions as a scaffold that facilitates the interaction between RNA-binding protein IGF2BP3 and *TGFBR1* mRNA, thereby enhancing its stability in an m^6^A-dependent manner. Consequently, *circTGFBR2(3-6)* promotes TGF-β-induced EMT, migration, extravasation, stemness, and chemotherapy resistance in breast cancer cells. Our findings highlight *circTGFBR2(3-6)* as a critical potentiator of TGF-β/SMAD signaling and a potential therapeutic target to modulate cancer cell plasticity.

## Results

### Characterization of *circTGFBR2(3-6)*, an enhancer of TGF-β/SMAD signaling

**Fig. 1:**
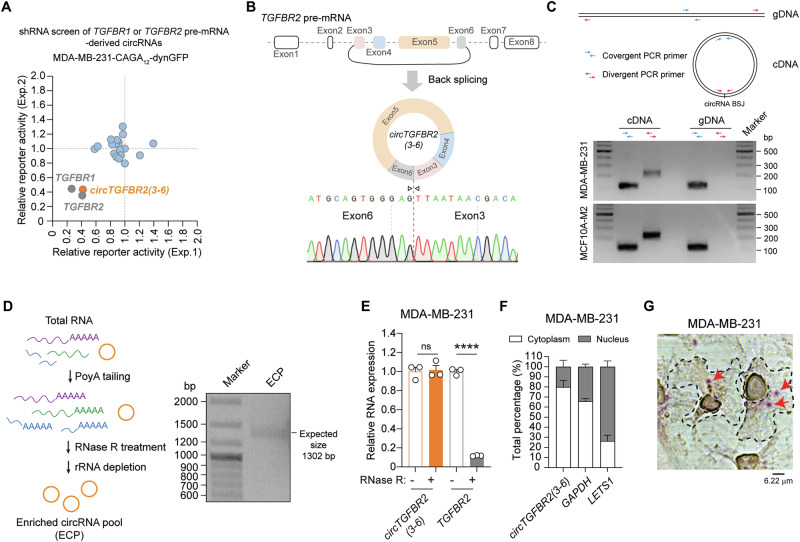
Characterization of *circTGFBR2(3-6)*, an activator of TGF-β/SMAD signaling. **A** Diagram showing the results of shRNA-mediated screening of *TGFBR1* or *TGFBR2* pre-mRNA-derived circRNAs in MDA-MB-231 cells stably expressing a SMAD3/4-driven (CAGA)_12_-dynGFP reporter in the presence of TGF-β (0.5 ng/mL). The *x-* and *y*-axes represent relative reporter activity from two independent experiments. shRNAs targeting linear *TGFBR1* and *TGFBR2* mRNA were taken along as control. **B** Illustration of *circTGFBR2(3-6)* biogenesis from *TGFBR2* pre-mRNA. Sanger sequencing confirmed the back-splicing junction (BSJ) sequence of *circTGFBR2(3-6)*. **C** PCR analysis of *circTGFBR2(3-6)* amplification from genomic DNA (gDNA) and complementary DNA (cDNA) of MDA-MB-231 and MCF10A-M2 cells, visualized by agarose gel electrophoresis. The schematic shows the positions and orientations of convergent and divergent PCR primers. **D** The workflow for preparing the MDA-MB-231-derived enriched circRNA pool (ECP) is illustrated on the left. The full-length *circTGFBR2(3-6)* PCR product, amplified from cDNA of the ECP, was analyzed by agarose gel electrophoresis (shown on the right). **E** RT-qPCR analysis of *circTGFBR2(3-6)* and *TGFBR2* mRNA expression in MDA-MB-231 cells following RNase R treatment. Data present mean ± SEM from three biological replicates. Statistical significance was calculated using one-way analysis of variance (ANOVA) followed by Tukey’s multiple comparisons test. **F** Subcellular localization analysis of *circTGFBR2(3-6)* in MDA-MB-231 cells by RT-qPCR. Long non-coding RNA *LETS1* [96] and *GAPDH* mRNA serve as nuclear and cytoplasmic markers, respectively. Data are presented as mean ± SEM from three biological replicates. **G** In situ hybridization analysis of *circTGFBR2(3-6)* subcellular localization using a probe specifically targeting its BSJ sequence in MDA-MB-231 cells. Scale bar = 6.22 μm. Cells and nuclei are outlined in black, and red arrows indicate *circTGFBR2(3-6)* signals.

We aimed to identify circRNAs derived from *TGFBR1* and *TGFBR2* pre-mRNAs that regulate TGF-β/SMAD signaling. To this end, we focused on 12 circRNAs from *TGFBR1* pre-mRNA and 6 circRNAs from *TGFBR2* pre-mRNA, based on circRNA annotation data from the TransCirc database [36]. We designed shRNAs specifically targeting their unique back-splicing junction (BSJ) sequence for selective depletion (Supplementary Fig. 1A). We performed a loss-of-function screen in MDA-MB-231 triple-negative breast cancer (TNBC) cells stably expressing a selective synthetic SMAD3/4-driven transcriptional reporter CAGA_12_-dynamic green fluorescent protein (dynGFP) [37]. The screening results demonstrated that knockdown of *circTGFBR2(3-6)*, but not the other circRNAs, suppressed the TGF-β-induced transcriptional response as potent as the blockage achieved by knockdown of *TGFBR1* or *TGFBR2* mRNA (Fig. 1A, Supplementary Fig. 1B). *circTGFBR2(3-6)* is derived from exons 3 to 6 of the *TGFBR2* pre-mRNA and has a length of 1302 nucleotides (nt) (Fig. 1B). The BSJ sequence of *circTGFBR2(3-6)* was experimentally validated by Sanger sequencing of the PCR product amplified with BSJ-spanning divergent primers (Fig. 1B, C). In addition, the divergent primers amplified *circTGFBR2(3-6)* from complementary DNA (cDNA) but not from genomic DNA (gDNA) in both MDA-MB-231 cells and MCF10A-M2 pre-malignant breast cells, thereby excluding the possibility that *circTGFBR2(3-6)* was generated by genomic rearrangements or PCR artifacts (Fig. 1C). We further validated the internal exon composition of *circTGFBR2(3-6)* by amplifying its full-length sequence from an enriched circRNA pool (ECP) derived from MDA-MB-231 cells (Fig. 1D). Sanger sequencing results confirmed that the complete sequence of *circTGFBR2(3-6)* matched *hsa_circ_0064654* (chr3:30686238-30715738) as annotated in the circBase database [38]. Compared to its linear counterpart, *TGFBR2* mRNA (detected by qPCR primers targeting *TGFBR2* exon2), *circTGFBR2(3-6)* was resistant to RNase R-mediated exonuclease digestion (Fig. 1E, Supplementary Fig. 1C), consolidating that *circTGFBR2(3-6)* is a circRNA. Subcellular fractionation followed by RT-qPCR demonstrated that *circTGFBR2(3-6)* was mainly localized in the cytoplasm of both MDA-MB-231 and MCF10A-M2 cells (Fig. 1F, Supplementary Fig. 1D). This result was confirmed by in situ hybridization using a probe specifically targeting the BSJ sequence (nts 1270-1313) of *circTGFBR2(3-6)* in MDA-MB-231 and non-small-cell lung adenocarcinoma A549 cells (Fig. 1G, Supplementary Fig. 1E).

### *circTGFBR2(3-6)* promotes TGF-β/SMAD signaling

**Fig. 2:**
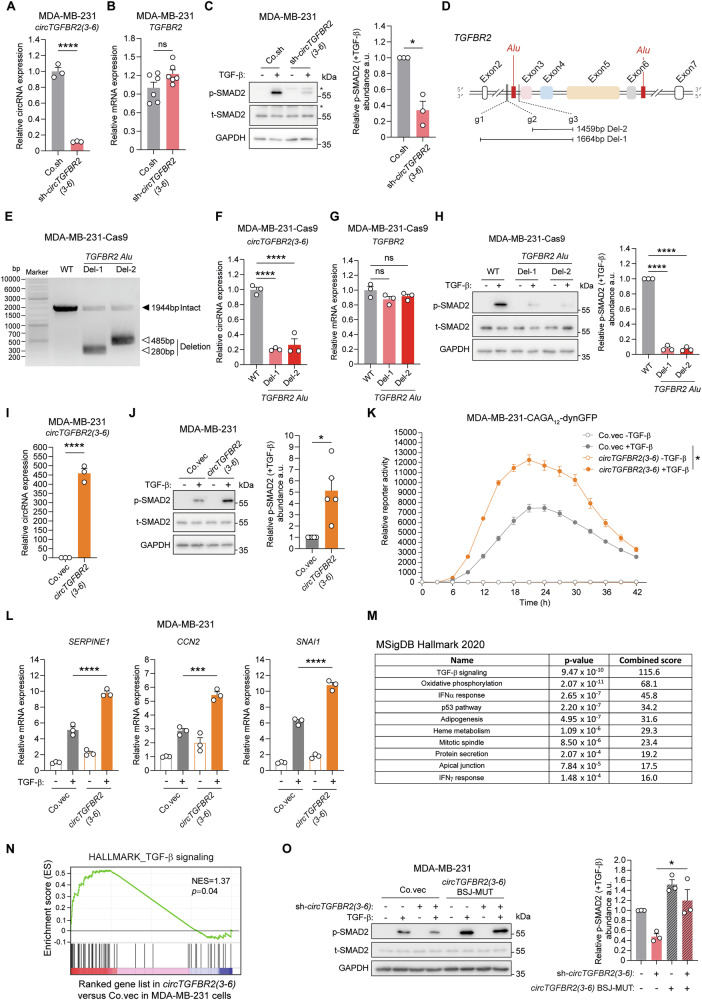
*circTGFBR2(3-6)* promotes TGF-β/SMAD signaling. RT-qPCR analysis of shRNA-mediated *circTGFBR2(3-6)* knockdown efficiency (**A**) and its effect on *TGFBR2* mRNA expression (**B**) in MDA-MB-231 cells. Data are presented as mean ± SEM from three (**A**) and six (**B**) biological replicates, respectively. Statistical significance was assessed using a two-tailed unpaired Student’s *t*-test. Co.sh, empty vector for shRNA expression. **C** Effect of shRNA-mediated *circTGFBR2(3-6)* knockdown on TGF-β-induced SMAD2 phosphorylation (p-SMAD2) response in MDA-MB-231 cells. GAPDH, loading control. An asterisk (*) indicates a non-specific band. Quantitative data represent the relative abundance of p-SMAD2 to total SMAD2 (t-SMAD2), expressed as mean ± SEM from three independent experiments. a.u. arbitrary units. Significance was assessed using a two-tailed paired Student’s *t*-test. **D** Schematic representation of the predicted *Alu* elements and the guide RNA (gRNA) positions used for *TGFBR2* intron2 genomic deletion (Del). **E** PCR analysis of the *TGFBR2* intron2 genomic region in wild-type (WT) and two MDA-MB-231 cell pools with *TGFBR2 Alu* deletion, visualized by agarose gel electrophoresis. RT-qPCR analysis of *TGFBR2 Alu* deletion-mediated *circTGFBR2(3-6)* knockdown efficiency (**F**) and its effect on *TGFBR2* mRNA expression (**G**) in MDA-MB-231 cells stably expressing Cas9. Data are presented as mean ± SEM from three biological replicates. Statistical significance was determined using one-way ANOVA followed by Dunnett’s multiple comparisons test. **H** Effect of genomic *TGFBR2 Alu* deletion-mediated *circTGFBR2(3-6)* knockdown on TGF-β-induced p-SMAD2 response in MDA-MB-231 cells stably expressing Cas9. Quantitative data represent p-SMAD2 levels relative to t-SMAD2, expressed as mean ± SEM from three independent experiments. GAPDH, loading control. Statistical significance was assessed using one-way ANOVA followed by Dunnett’s multiple comparisons test. **I** RT-qPCR analysis of *circTGFBR2(3-6)* ectopic expression efficiency in MDA-MB-231 cells. Data are shown as mean ± SEM from three biological replicates. Statistical significance was determined using a two-tailed unpaired Student’s *t*-test. Co.vec empty vector control. **J** Effect of *circTGFBR2(3-6)* ectopic expression on TGF-β-induced p-SMAD2 levels in MDA-MB-231 cells. Quantitative data represent p-SMAD2 abundance relative to t-SMAD2, shown as mean ± SEM from five independent experiments. GAPDH, loading control. Statistical significance was assessed using a two-tailed paired Student’s *t*-test. **K** Effect of *circTGFBR2(3-6)* ectopic expression on the TGF-β-induced CAGA_12_-dynGFP reporter activity in MDA-MB-231 cells. Statistical significance was assessed using one-way ANOVA followed by Dunnett’s multiple comparisons test. Data are presented as mean ± SEM from six biological replicates. **L** RT-qPCR analysis of the effect of *circTGFBR2(3-6)* ectopic expression on TGF-β-induced target gene (*SERPINE1*, *CCN2*, and *SNAI1*) expression in MDA-MB-231 cells. Data are shown as mean ± SEM from three biological replicates. Statistical significance was assessed using one-way ANOVA followed by Tukey’s multiple comparisons test. **M** List of significantly changed pathways affected by *circTGFBR2(3-6)* ectopic expression in MDA-MB-231 cells. **N** GSEA revealed a positive correlation between (manipulated) *circTGFBR2(3-6)* expression and the TGF-β response gene signature. NES, normalized enrichment score. **O** Effect of shRNA-resistant *circTGFBR2(3-6)* overexpression on TGF-β-induced p-SMAD2 response in MDA-MB-231 cells upon shRNA-mediated endogenous *circTGFBR2(3-6)* knockdown. Quantitative data show p-SMAD2 abundance relative to t-SMAD2, expressed as mean ± SEM from three independent experiments. GAPDH, loading control. Statistical significance was assessed using one-way ANOVA followed by Tukey’s multiple comparisons test.

We continued studying the effect of *circTGFBR2(3-6)* on TGF-β/SMAD signaling. shRNA-mediated selective depletion of *circTGFBR2(3-6)*, without targeting the linear *TGFBR2* mRNA, inhibited TGF-β-induced SMAD2 phosphorylation (p-SMAD2) levels, which is upstream of the TGF-β-induced transcriptional response [11, 12], in MDA-MB-231 cells, MCF10A normal breast cells, and MCF10A-M2 cells (Fig. 2A–C, Supplementary Fig. 2A, B). To eliminate the off-target effects from the shRNA, we employed an orthogonal approach to knockdown *circTGFBR2(3-6)* by disrupting its adjacent genomic sequences responsible for back-splicing [39]. Using pairwise sequence alignment (PSA) analysis [40], we predicted two putative inverted *Alu* retroelements, which are required for circRNA biogenesis [39], within the exon-flanking genomic sequences of *TGFBR2* exon3-6 (Fig. 2D, Supplementary Table 1). The clustered regularly interspaced palindromic repeats (CRISPR)–CRISPR-associated protein 9 (Cas9) system with two combinations of paired guide (g)RNAs (g1 + g3 and g2 + g3) was utilized to delete a ~1500 bp genomic fragment, which contains the putative *Alu* element and its flanking DNA sequences, in *TGFBR2* intron2 (Fig. 2D). PCR analysis confirmed a genomic deletion in a pool of MDA-MB-231-Cas9 cells transduced with paired gRNAs (Del-1 and Del-2), compared to those transduced with empty vector control (WT) (Fig. 2E). As expected, genomic deletion of the TGFBR2 *Alu* element led to a decrease in *circTGFBR2(3-6)* expression, while *TGFBR2* mRNA expression remained unaffected (Fig. 2F, G). Consistent with the shRNA-mediated effect, *circTGFBR2(3-6)* knockdown by *Alu* deletion inhibited TGF-β-induced p-SMAD2 response in MDA-MB-231 cells (Fig. 2H). To further complement these results, an expression vector with flanking complementary *Alu* minimal elements to facilitate circularization [41] was employed to ectopically express *circTGFBR2(3-6)* in MDA-MB-231 cells (Fig. 2I). Sanger sequencing confirmed that no unwanted vector sequences were incorporated into the BSJ sequence of the overexpressed *circTGFBR2(3-6)* (Supplementary Fig. 2C). We found that TGF-β-induced p-SMAD2 levels were promoted by ectopic expression of *circTGFBR2(3-6)*, but not by its linear counterpart, *TGFBR2 exon3-6*, in MDA-MB-231 cells (Fig. 2J, Supplementary Fig. 2D, E). In addition, the TGF-β-induced transcriptional activity and the expression of TGF-β target genes (i.e., *SERPINE1*, *CCN2*, and *SNAI1*) were induced to higher levels in MDA-MB-231 cells with *circTGFBR2(3-6)* overexpression as compared to control cells (Fig. 2K, L). Moreover, pathway enrichment analysis [42] of *circTGFBR2(3-6)*-induced genes, identified by whole-transcriptome RNA-seq, revealed TGF-β signaling as the most significantly impacted cellular pathway (Fig. 2M). Gene set enrichment analysis (GSEA) confirmed a positive correlation between manipulated *circTGFBR2(3-6)* expression and the TGF-β response gene signature (Fig. 2N). To further validate our results, rescue experiments were performed in MDA-MB-231 cells by ectopic expression of *circTGFBR2(3-6)* carrying a mutated BSJ (BSJ-MUT), rendering it resistant to shRNA targeting (Supplementary Fig. 2F, G). *circTGFBR2(3-6)* BSJ-MUT ectopic expression restored the inhibitory effect of endogenous *circTGFBR2(3-6)* knockdown on TGF-β-induced p-SMAD2 levels (Fig. 2O). Taken together, these results suggest that *circTGFBR2(3-6)* potentiates TGF-β/SMAD signaling in breast cancer cells.

### *circTGFBR2(3-6)* promotes TGF-β-induced cellular responses including EMT and migration

**Fig. 3:**
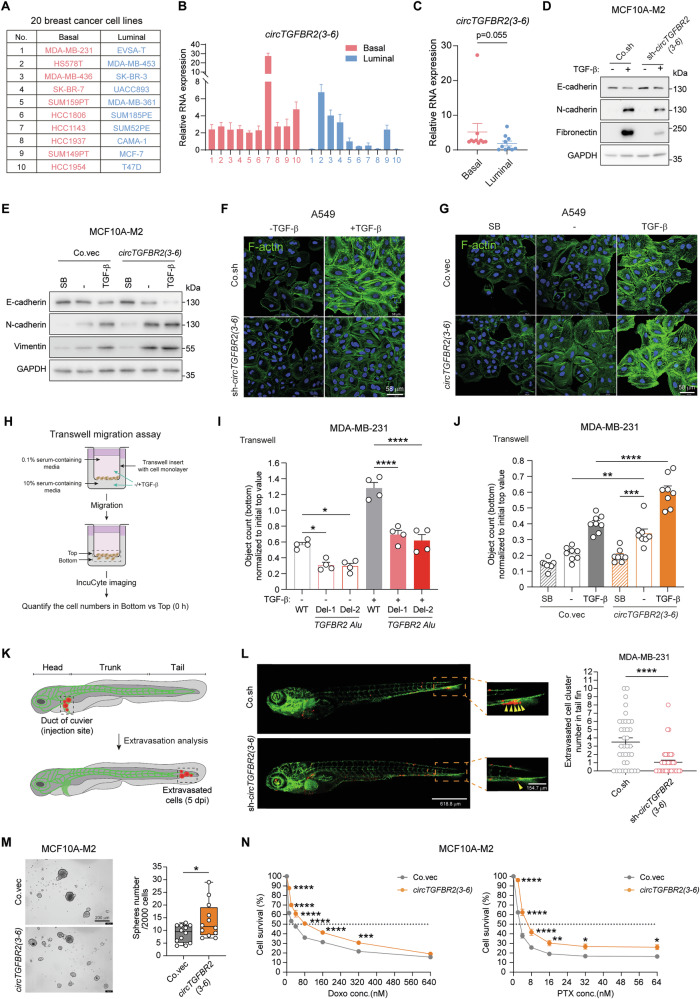
*circTGFBR2(3-6)* promotes TGF-β-induced EMT, migration, extravasation, stemness, and chemotherapy resistance. **A** List of 20 breast cancer cell lines used to compare the expression of genes of interest between luminal and basal subtypes in this study. **B** RT-qPCR analysis of *circTGFBR2(3-6)* expression across the 20 breast cancer cell lines. Data are presented as mean ± SEM from three technical replicates. **C** Comparison of *circTGFBR2(3-6)* expression between basal-type and luminal-type breast cancer cell lines shown in (**B**). Statistical significance was assessed using a two-tailed unpaired Student’s *t*-test. Effect of shRNA-mediated *circTGFBR2(3-6)* knockdown (**D**) and *circTGFBR2(3-6)* ectopic expression (**E**) on TGF-β-induced EMT marker expression in MCF10A-M2 cells. GAPDH, loading control. Immunofluorescence analysis of F-actin expression and localization in A549 cells upon shRNA-mediated *circTGFBR2(3-6)* knockdown (**F**) and *circTGFBR2(3-6)* ectopic expression (**G**), in the absence or presence of TGF-β or SB505124 (SB). Scale bar = 58 μm. **H** Schematic representation of the transwell migration assay. Effect of *circTGFBR2(3-6)* on TGF-β-induced migration in MDA-MB-231 cells, analyzed using a transwell migration assay. *circTGFBR2(3-6)* knockdown was achieved by genomic *TGFBR2 Alu* deletion (**I**), and *circTGFBR2(3-6)* overexpression was achieved by ectopic expression (**J**). Data are presented as mean ± SEM from four (**I**) and eight (**J**) biological replicates, respectively. Statistical significance was calculated using one-way ANOVA followed by Tukey’s multiple comparisons test. **K** Schematic representation of the zebrafish embryo xenograft assay. **L** In vivo zebrafish embryo xenograft experiments with mCherry-labeled MDA-MB-231 cells upon shRNA-mediated *circTGFBR2(3-6)* knockdown. Extravasated breast cancer cell clusters are indicated by yellow arrows. Analysis of the extravasated cell cluster numbers is expressed as mean ± SEM (n = 36 in gEV group and n = 41 in sh-*circTGFBR2(3-6)* group). Statistical significance was assessed using a two-tailed unpaired Student’s *t*-test. Whole zebrafish image, scale bar = 618.8 μm; zoomed image, scale bar = 154.7 μm. **M** Effect of *circTGFBR2(3-6)* ectopic expression on mammosphere formation in MCF10A-M2 cells. Scale bar = 200 μm. The results are quantified as a box plot with min to max Whiskers from 12 biological replicates, with significance analyzed using a two-tailed unpaired Student’s *t*-test. **N** Dose-response curves for doxorubicin (Doxo) and paclitaxel (PTX) in MCF10A-M2 cells upon *circTGFBR2(3-6)* ectopic expression. Data are presented as mean ± SEM from three biological replicates. Statistical significance was assessed using two-way ANOVA followed by Šídák’s multiple comparisons test.

To assess the relationship between *circTGFBR2(3-6)* expression and breast cancer progression, we analyzed its expression in a panel of 20 breast cancer cell lines, comprising 10 aggressive basal-type and 10 less aggressive luminal-type cell lines [43] (Fig. 3A). *circTGFBR2(3-6)* was more highly expressed (p = 0.055) in the basal-type cell lines than in luminal-type ones (Fig. 3B, C). Given that *circTGFBR2(3-6)* and *TGFBR2* originate from the same pre-mRNA, *TGFBR2* mRNA and protein levels were also upregulated in the basal-type cell lines compared to luminal-type cell lines (Supplementary Fig. 3A, B). To investigate the role of *circTGFBR2(3-6)* in TGF-β-induced EMT, we depleted it using shRNA in epithelial MCF10A-M2 cells (Supplementary Fig. 3C). Western blotting analysis demonstrated that *circTGFBR2(3-6)* knockdown mitigated TGF-β-induced downregulation of the epithelial marker E-cadherin and upregulation of mesenchymal markers, including N-cadherin and Fibronectin (Fig. 3D). Conversely, *circTGFBR2(3-6)* ectopic expression potentiated the TGF-β-induced changes in EMT marker expression in MCF10A-M2 cells (Fig. 3E, Supplementary Fig. 3D). Of note, blocking TGF-β signaling with a selective small-molecule TGFBR1 kinase inhibitor SB505124 [44] (SB) abolished *circTGFBR2(3-6)*-triggered changes in EMT marker expression, indicating that TGF-β signaling activation is essential for *circTGFBR2(3-6)*-mediated EMT (Fig. 3E). In agreement with these results, *circTGFBR2(3-6)* depletion suppressed, whereas its overexpression promoted, TGF-β-induced filamentous actin (F-actin) stress fiber formation in A549 cells, a widely used model for studying TGF-β-induced EMT [45] (Fig. 3F, G, Supplementary Fig. 3E, F). Additionally, *circTGFBR2(3-6)* knockdown by shRNA or genomic *Alu* deletion inhibited, while its ectopic expression enhanced, TGF-β-induced migration, as measured by transwell migration assays (Fig. 3H–J, Supplementary Fig. 3G). An in vivo zebrafish xenograft cancer model [46] was employed to further consolidate these findings (Fig. 3K). shRNA-mediated *circTGFBR2(3-6)* depletion significantly impaired the extravasation ability of MDA-MB-231 cells in zebrafish embryos (Fig. 3L). Given that TGF-β signaling confers stemness [47] and chemotherapy resistance [48, 49] to cancer cells, we next evaluated the mammosphere-forming capability of MCF10A-M2 cells following *circTGFBR2(3-6)* ectopic expression. As expected, *circTGFBR2(3-6)* facilitated mammosphere formation of MCF10A-M2 cells (Fig. 3M). Moreover, *circTGFBR2(3-6)* overexpression enhanced the resistance of MCF10A-M2 cells to the chemotherapeutic drugs doxorubicin (Doxo) and paclitaxel (PTX) (Fig. 3N). We further extended our findings by assessing the effect of *circTGFBR2(3-6)* on TGF-β-induced inhibition of proliferation/viability in normal human keratinocyte HACAT cells. In this context, *circTGFBR2(3-6)* knockdown mitigated TGF-β-induced inhibition of cell proliferation/viability (Supplementary Fig. 3H). Moreover, *circTGFBR2(3-6)* depletion decreased TGF-β-induced expression of the cell cycle progression inhibitory genes *CDKN2B* (encoding p15) and *CDKN1A* (encoding p27), as well as the canonical target gene *SERPINE1* (Supplementary Fig. 3I). Taken together, our data suggest that *circTGFBR2(3-6)* enhances TGF-β-induced cellular responses including EMT, migration, extravasation, stemness, and chemotherapeutic drug resistance in breast cancer cells, while promoting TGF-β-induced cytostatic effect in normal cells.

### *circTGFBR2(3-6)* binds to and stabilizes *TGFBR1* mRNA

**Fig. 4:**
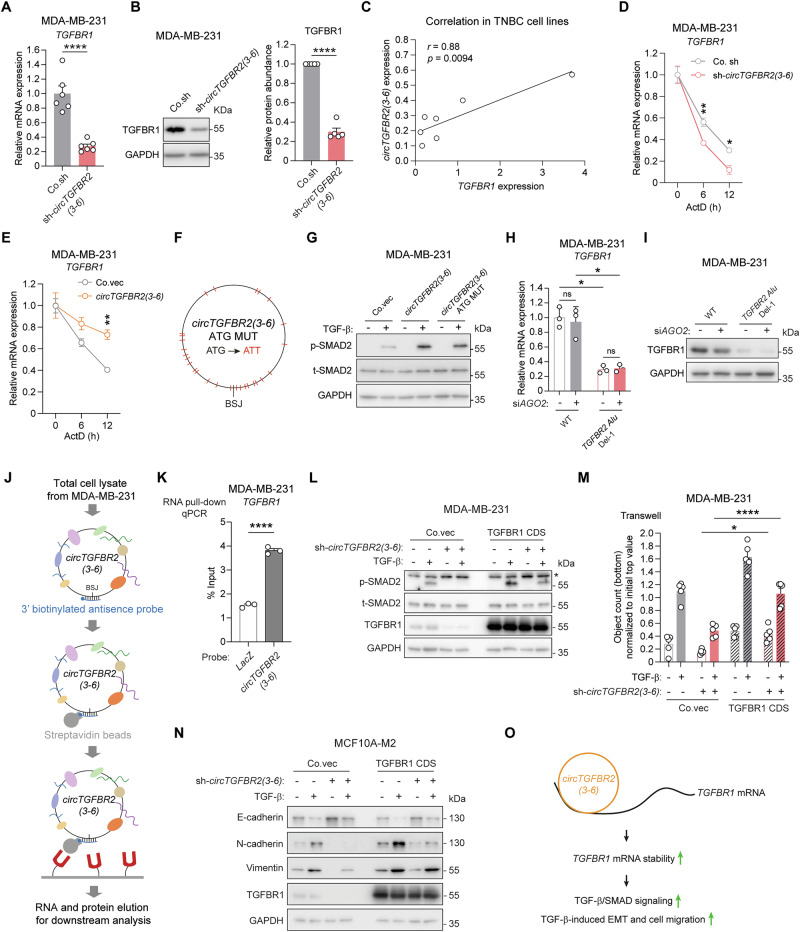
*circTGFBR2(3-6)* binds to and stabilizes *TGFBR1* mRNA. **A** RT-qPCR analysis of *TGFBR1* mRNA expression in MDA-MB-231 cells upon shRNA-mediated *circTGFBR2(3-6)* knockdown. Data are presented as the mean ± SEM from six biological replicates, with significance assessed using a two-tailed unpaired Student’s *t*-test. **B** Effect of shRNA-mediated *circTGFBR2(3-6)* knockdown on TGFBR1 protein expression in MDA-MB-231 cells. GAPDH, loading control. Data are presented as the mean ± SEM from five independent experiments, with significance assessed using a two-tailed paired Student’s *t*-test. **C** Correlation between *circTGFBR2(3-6)* and *TGFBR1* mRNA expression across seven triple-negative breast cancer (TNBC) cell lines. Pearson’s *r* and two-tailed *p* value were used to assess correlation. *TGFBR1* mRNA stability upon shRNA-mediated *circTGFBR2(3-6)* knockdown (**D**) or *circTGFBR2(3-6)* ectopic expression (**E**), measured by time-course experiments using actinomycin D (ActD). RT-qPCR data are presented as the mean ± SEM from three biological replicates, with significance assessed using multiple unpaired *t*-tests. **F** Schematic representation of a *circTGFBR2(3-6)* mutant in which all ATG codons mutated to ATT codons (*circTGFBR2(3-6)* ATG MUT). **G** Effect of *circTGFBR2(3-6)* and *circTGFBR2(3-6)* ATG MUT ectopic expression on TGF-β-induced p-SMAD2 response in MDA-MB-231 cells. Quantitative of p-SMAD2 abundance relative to t-SMAD2 is shown as mean ± SEM from three independent experiments. GAPDH, loading control. Significance was calculated using one-way ANOVA followed by Dunnett’s multiple comparisons test. RT-qPCR (**H**) and Western blotting (**I**) analysis of *TGFBR1* mRNA and protein expression in MDA-MB-231 cells (WT and *TGFBR2 Alu* Del-1) upon siRNA-mediated *AGO2* knockdown. GAPDH, loading control. Data in (**H**) are expressed as mean ± SEM from three biological replicates, with significance assessed using two-way ANOVA followed by uncorrected Fisher’s LSD test. **J** Schematic representation of RNA pull-down of *circTGFBR2(3-6)*. **K** Interaction between *circTGFBR2(3-6)* and *TGFBR1* mRNA in MDA-MB-231 cells, analyzed by RNA pull-down followed by RT-qPCR. Data represent mean ± SEM from three biological replicates, with significance assessed using a two-tailed unpaired Student’s *t*-test. A *LacZ*-targeting probe served as a control. **L** Effect of TGFBR1 CDS ectopic expression on TGF-β-induced p-SMAD2 response in MDA-MB-231 cells upon shRNA-mediated *circTGFBR2(3-6)* knockdown. An asterisk (*) indicates a non-specific band. GAPDH, loading control. **M** Effect of TGFBR1 CDS ectopic expression on MDA-MB-231 cell migration upon shRNA-mediated *circTGFBR2(3-6)* knockdown, measured using a transwell assay. Data are presented as mean ± SEM from five biological replicates. Significance was assessed using two-way ANOVA followed by Tukey’s multiple comparisons test. **N** Effect of TGFBR1 CDS ectopic expression on TGF-β-induced EMT marker expression in MCF10A-M2 cells upon shRNA-mediated *circTGFBR2(3-6)* knockdown. GAPDH, loading control. **O** Proposed working model. *circTGFBR2(3-6)* interacts with and stabilizes *TGFBR1* mRNA, resulting in the enhancement of TGF-β/SMAD signaling, as well as TGF-β-induced EMT and cell migration.

Next, we sought to elucidate the mechanism by which *circTGFBR2(3-6)* promotes TGF-β/SMAD signaling. Given the finding that TGF-β-induced p-SMAD2 response, a direct indicator of TGF-β receptor activity, was enhanced by *circTGFBR2(3-6)* (Fig. 2J), we examined the expression of *TGFBR1* mRNA. We demonstrated that *TGFBR1* mRNA expression was downregulated in MDA-MB-231 cells upon *circTGFBR2(3-6)* depletion using multiple approaches, including shRNA, siRNA, and genomic *Alu* deletion (Fig. 4A, Supplementary Fig. 4A, B). Moreover, we observed a reduction in TGFBR1 protein expression upon *circTGFBR2(3-6)* knockdown (Fig. 4B). These results were further confirmed in MCF10A-M2 cells (Supplementary Fig. 4C, D). On the contrary, *TGFBR1* mRNA and protein levels were enhanced in MDA-MB-231 cells following *circTGFBR2(3-6)* overexpression (Supplementary Fig. 4E, F). However, knockdown of *TGFBR2* mRNA using two independent shRNA constructs targeting its 3′ untranslated region (3′UTR) did not change the expression of *circTGFBR2(3-6)* or *TGFBR1* mRNA in MDA-MB-231 cells (Supplementary Fig. 4G). Importantly, the expression of *circTGFBR2(3-6)* and *TGFBR1* mRNA showed a positive correlation across seven TNBC cell lines (Fig. 4C, Supplementary Fig. 4H). Similarly, *TGFBR2* mRNA expression positively correlated with both *circTGFBR2(3-6)* and *TGFBR1* mRNA expression across this panel of TNBC cell lines (Supplementary Fig. 4H, I). Given that *circTGFBR2(3-6)* is localized in the cytoplasm (Fig. 1G), we hypothesized that it might protect *TGFBR1* mRNA from degradation. Consistent with this assumption, time-course experiments using the transcription inhibitor actinomycin D (ActD) [50] revealed that *circTGFBR2(3-6)* enhanced *TGFBR1* mRNA stability in MDA-MB-231 cells (Fig. 4D, E).

circRNAs can encode small peptides to elicit their effects [32, 33]. To exclude this possibility, we generated a *circTGFBR2(3-6)* mutant whose putative start codons were mutated (from ATG to ATT; Fig. 4F). TGF-β-induced p-SMAD2 response was enhanced to a similar extent by both ectopic expression of *circTGFBR2(3-6)* and its ATG mutant in MDA-MB-231 cells, suggesting that *circTGFBR2(3-6)* functions as a non-coding circRNA to promote TGF-β/SMAD signaling (Fig. 4G, Supplementary Fig. 4J, K). circRNAs can facilitate the expression of their target mRNAs by sponging miRNAs [30, 31]. To rule out the involvement of miRNAs, we depleted *Argonaute2* (*AGO2*), which encodes the core catalytic component of the RNA-induced silencing complex (RISC) [51], using a pool of siRNAs in MDA-MB-231 cells (Supplementary Fig. 4L). However, *AGO2* knockdown did not reverse the inhibitory effect of *circTGFBR2(3-6)* knockdown on *TGFBR1* mRNA and protein expression (Fig. 4H, I, Supplementary Fig. 4L, M). Next, we investigated whether *circTGFBR2(3-6)* directly binds to *TGFBR1* mRNA to regulate its stability. To selectively capture *circTGFBR2(3-6)*, we designed a biotinylated antisense probe targeting its BSJ sequence (Fig. 4J). RNA pull-down followed by RT-qPCR revealed that *TGFBR1* mRNA was enriched together with *circTGFBR2(3-6)* in lysates from MDA-MB-231 cells, suggesting an interaction between these two RNA molecules (Fig. 4J, K, Supplementary Fig. 4N). Linear *TGFBR2* mRNA was not enriched, confirming the specificity of the BSJ-targeting probe (Supplementary Fig. 4N). To evaluate whether *circTGFBR2(3-6)* exerts its effects by regulating *TGFBR1* mRNA expression, we performed rescue experiments using ectopic expression of the *TGFBR1* coding sequence (CDS). *TGFBR1* CDS overexpression restored TGF-β-induced p-SMAD2 levels and cell migration response in MDA-MB-231 cells (Fig. 4L, M), as well as the changes in TGF-β-induced EMT marker expression in MCF10A-M2 cells (Fig. 4N), which had been inhibited by *circTGFBR2(3-6)* knockdown. Taken together, these findings suggest that *circTGFBR2(3-6)* binds to and stabilizes *TGFBR1* mRNA, thereby promoting TGF-β/SMAD signaling and enhancing TGF-β-induced EMT and cell migration (Fig. 4O).

### *circTGFBR2(3-6)* scaffolds RNA-binding protein IGF2BP3 and *TGFBR1* mRNA

**Fig. 5:**
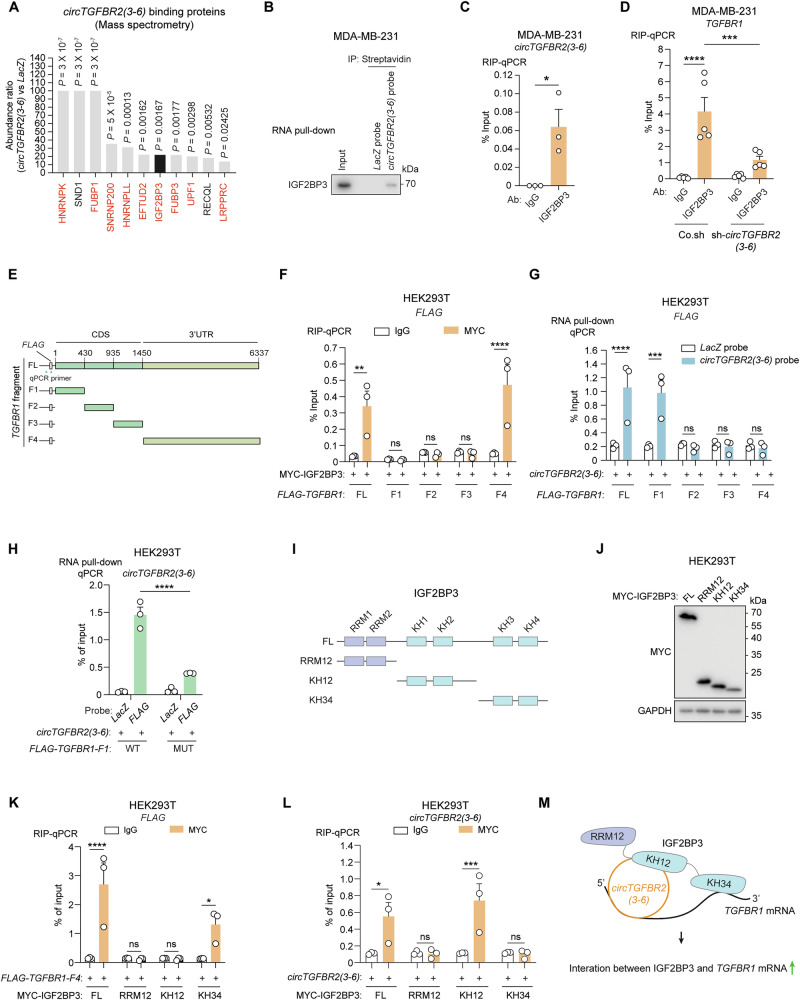
*circTGFBR2(3-6)* scaffolds IGF2BP3 and *TGFBR1* mRNA. **A** Identification of *circTGFBR2(3-6)*-binding proteins by RNA pull-down followed by mass spectrometry. The top statistically significant hits are shown. RNA-binding proteins are marked in red. **B** RNA pull-down analysis of *circTGFBR2(3-6)*-IGF2BP3 interaction in MDA-MB-231 cells. Western blotting with an IGF2BP3 antibody was used to detect IGF2BP3 in whole-cell lysates (Input) and immunoprecipitates (IP). **C** RNA immunoprecipitation (RIP) analysis confirming *circTGFBR2(3-6)*-IGF2BP3 interaction in MDA-MB-231 cells. RT-qPCR was performed to quantify *circTGFBR2(3-6)* levels in immunoprecipitates. Data are presented as mean ± SEM from three biological replicates, with significance assessed using an unpaired Student’s *t*-test. **D** RIP assay assessing *TGFBR1* mRNA-IGF2BP3 interaction upon shRNA-mediated *circTGFBR2(3-6)* knockdown in MDA-MB-231 cells. RT-qPCR was performed to detect *TGFBR1* in immunoprecipitates. Data represent mean ± SEM from five independent experiments, with significance assessed using two-way ANOVA followed by uncorrected Fisher’s LSD test. **E** Schematic representation of full-length *TGFBR1* (FL) and its truncation mutants (F1, F2, F3, and F4). Locations of CDS and 3′UTR are indicated. **F** RIP assay evaluating the interaction between *FLAG-TGFBR1* mRNA (FL and truncation mutants) and MYC-IGF2BP3 in HEK293T cells. RT-qPCR detected *FLAG* in immunoprecipitates. Data represent mean ± SEM from three biological replicates, with significance analyzed using two-way ANOVA followed by Šídák’s multiple comparisons test. **G** RNA pull-down assay assessing the interaction between *FLAG-TGFBR1* mRNA (FL and truncation mutants) and *circTGFBR2(3-6)* in HEK293T cells. RT-qPCR detected *FLAG* in immunoprecipitates. Data represented as mean ± SEM from three biological replicates, with significance assessed using two-way ANOVA followed by Šídák’s multiple comparisons test. **H** Interaction between *circTGFBR2(3-6)* and either WT or MUT *FLAG-TGFBR1-F1* in HEK293T cells, analyzed by RNA pull-down followed by RT-qPCR. Data represent mean ± SEM from three biological replicates, with significance assessed using a two-tailed unpaired Student’s *t*-test. **I** Schematic representation of FL IGF2BP3 and its truncation mutants (RRM12, KH12, and KH34). **J** Western blotting analysis of MYC-IGF2BP3 and its truncation mutants in HEK293T cells. GAPDH, loading control. RIP assay assessing the interaction between MYC-IGF2BP3 FL or truncation mutants and *FLAG-TGFBR1-F4* (**K**) or *circTGFBR2(3-6)* (**L**) and in HEK293T cells. RT-qPCR detected *FLAG* in immunoprecipitates. Data are presented as mean ± SEM from three biological replicates, with significance analyzed using two-way ANOVA followed by Šídák’s multiple comparisons test. **M** Schematic working model for how *circTGFBR2(3-6)* binds to the KH12 di-domain of IGF2BP3 and promotes its interaction with *TGFBR1* mRNA through the KH34 di-domain.

To identify protein partners of *circTGFBR2(3-6)* that are involved in its effect on *TGFBR1* mRNA stabilization, we performed RNA pull-down followed by mass spectrometry analysis using lysates from MDA-MB-231 cells (Fig. 4J). Interactome analysis showed that the proteins most enriched using the *circTGFBR2(3-6)* probe, relative to the *LacZ* control probe, were predominantly RNA-binding proteins (Fig. 5A, Supplementary Fig. 5A). We focused on IGF2BP3, a well-characterized pro-tumorigenic RNA-binding protein and m^6^A reader [52, 53], for further investigation (Fig. 5A, Supplementary Fig. 5A). RNA pull-down analysis and RNA immunoprecipitation (RIP) followed by RT-qPCR confirmed the interaction between *circTGFBR2(3-6)* and IGF2BP3 (Fig. 5B, C). We hypothesized that IGF2BP3 mediates the effects of *circTGFBR2(3-6)* by binding to *TGFBR1* mRNA. As expected, IGF2BP3 interacted with *TGFBR1* mRNA, and this interaction was reduced upon *circTGFBR2(3-6)* knockdown in MDA-MB-231 cells (Fig. 5D). These results suggest that *circTGFBR2(3-6)* may function as a scaffold for IGF2BP3 protein and *TGFBR1* mRNA. To map the binding regions of IGF2BP3 protein and *circTGFBR2(3-6)* on *TGFBR1* mRNA, we generated mRNA truncation mutants from the *TGFBR1* CDS and 3′UTR, fused with the *FLAG* epitope tag sequence (Fig. 5E). The expression of ectopically expressed *TGFBR1* fragments was specifically detected by qPCR primers spanning the *FLAG* sequence (Fig. 5E). RIP-qPCR results showed that only the fragment 4 (F4), which contains the *TGFBR1* 3′UTR sequence, co-precipitated with MYC-tagged IGF2BP3 in HEK293T cells, whereas the other fragments did not (Fig. 5F). However, *circTGFBR2(3,6)* interacted with fragment 1 (F1), a 430-nt sequence located in the 5′ region of the *TGFBR1* CDS (Fig. 5G). Furthermore, the base-pairing prediction tool IntaRNA [54] identified a putative interaction region between *circTGFBR2(3-6)* and *TGFBR1* mRNA (Supplementary Fig. 5B). Mutation of this base-pairing region in the *TGFBR1-F1* fragment reduced its binding to *circTGFBR2(3-6)* (Fig. 5H). Notably, the *circTGFBR2(3-6*) BSJ-MUT retained its ability to bind *TGFBR1-F1* as efficiently as the wild-type *circTGFBR2(3-6*) (Supplementary Fig. 5C).

Next, we analyzed the IGF2BP3 domains responsible for binding to *TGFBR1* mRNA and *circTGFBR2(3-6)*. We generated IGF2BP3 truncation mutants by dividing the protein into three segments: the N-terminal RNA recognition motif (RRM) di-domain (RRM12) and two C-terminal K homology (KH) di-domains (KH12 and KH34), each comprising two tandem RNA-binding domains [19, 55] (Fig. 5I, J). RIP-qPCR results revealed that the KH34 di-domain specifically bound to the *TGFBR1* 3′UTR fragment F4, while the KH12 di-domain interacted with *circTGFBR2(3-6)* (Fig. 5K, L). Furthermore, we identified two regions within *circTGFBR2(3-6)* that contain the RNA consensus sequence CA-N_15-25_-CGGCA, which is selectively recognized by the KH12 di-domain of IGF2BP3 [56] (Supplementary Fig. 5D). In vitro RNA pull-down assays confirmed that the purified IGF2BP3-KH12 protein directly interacted with RNA probes containing either region 1 or region 2 of *circTGFBR2(3-6)* (Supplementary Fig. 5E, F). Collectively, these findings suggest that *circTGFBR2(3-6)* associates with the KH12 di-domain of IGF2BP3 to promote binding of the *TGFBR1* 3′UTR to the KH34 di-domain of IGF2BP3 (Fig. 5M).

### IGF2BP3 is a key effector of *circTGFBR2(3-6)* to promote TGF-β/SMAD signaling

**Fig. 6:**
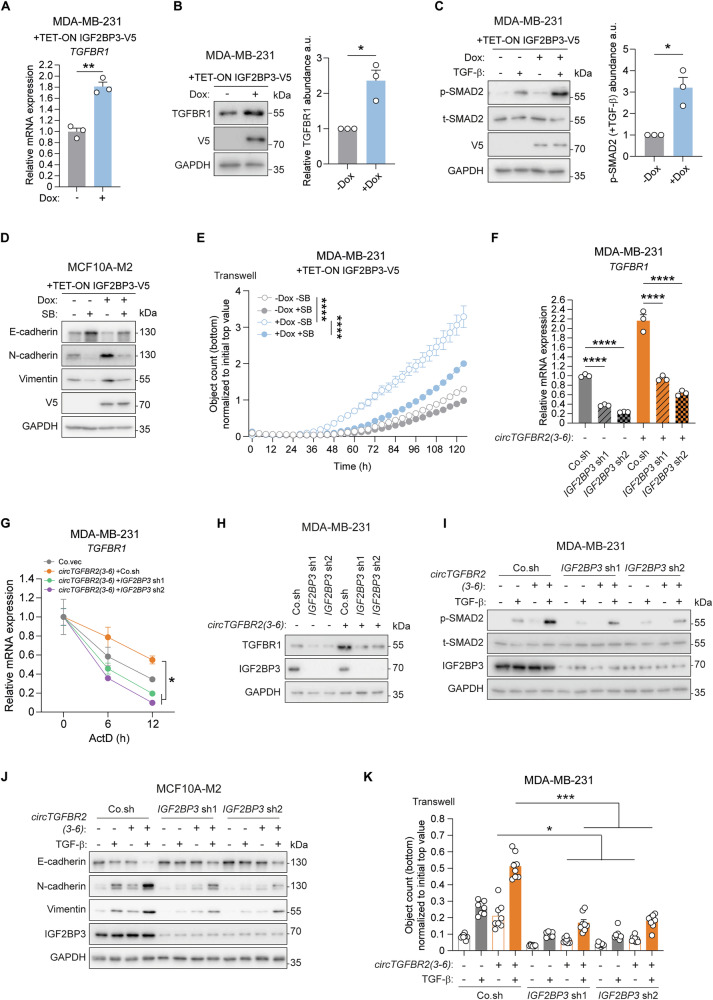
IGF2BP3 is a key effector of *circTGFBR2(3-6)* in promoting TGF-β/SMAD signaling*.* RT-qPCR (**A**) and Western blotting (**B**) analysis of *TGFBR1* mRNA and protein expression in MDA-MB-231 cells upon IGF2BP3 ectopic expression using a TET-ON inducible system. GAPDH, loading control. Data are presented as mean ± SEM from three biological replicates (**A**) and three independent experiments (**B**), with significance analyzed using a two-tailed unpaired (**A**) and paired (**B**) Student’s *t*-test, respectively. **C** Effect of IGF2BP3 ectopic expression (using a TET-ON inducible system) on TGF-β-induced p-SMAD2 in MDA-MB-231 cells. Quantitative data show the abundance of p-SMAD2 relative to t-SMAD2 (mean ± SEM from three independent experiments), with significance calculated using a two-tailed paired Student’s *t*-test. GAPDH, loading control. **D** Effect of SB505124 (SB) on EMT marker expression in MCF10A-M2 cells upon IGF2BP3 ectopic expression using a TET-ON inducible system. GAPDH, loading control. **E** Effect of SB505124 (SB) on MDA-MB-231 cell migration upon IGF2BP3 ectopic expression using a TET-ON inducible system, measured using a transwell assay for the indicated time. Data are presented as mean ± SEM from nine biological replicates, with significance assessed using two-way ANOVA followed by Tukey’s multiple comparisons test. **F** RT-qPCR analysis of *TGFBR1* mRNA expression in MDA-MB-231 cells upon *IGF2BP3* knockdown and *circTGFBR2(3-6)* ectopic expression. Data are presented as mean ± SEM from three biological replicates, with significance assessed using one-way ANOVA followed by Tukey’s multiple comparisons test. **G** Effect of *IGF2BP3* knockdown and *circTGFBR2(3-6)* ectopic expression on *TGFBR1* mRNA stability, measured in a time-course experiment using actinomycin D (ActD) in MDA-MB-231 cells. Data are presented as mean ± SEM from three biological replicates, with significance analyzed using two-way ANOVA followed by Dunnett’s multiple comparisons test. **H** Western blotting analysis of TGFBR1 protein expression in MDA-MB-231 cells upon *IGF2BP3* knockdown and *circTGFBR2(3-6)* ectopic expression. GAPDH, loading control. Effect of *IGF2BP3* knockdown and *circTGFBR2(3-6)* ectopic expression on TGF-β-induced p-SMAD2 levels in MDA-MB-231 cells (**I**) and TGF-β-induced EMT maker expression in MCF10A-M2 cells (**J**). GAPDH, loading control. **K** Effect of *IGF2BP3* knockdown and *circTGFBR2(3-6)* ectopic expression on TGF-β-induced MDA-MB-231 cell migration, measured using a transwell migration assay. Data are presented as mean ± SEM from eight biological replicates, with significance analyzed using two-way ANOVA followed by Tukey’s multiple comparisons test.

Given that IGF2BP3 enhances target mRNA stability by protecting them from degradation [19, 52, 53], we investigated whether its binding increases *TGFBR1* mRNA expression. As expected, IGF2BP3 ectopic expression using a doxycycline (Dox)-inducible TET-ON system elevated both *TGFBR1* mRNA and protein levels in MDA-MB-231 cells (Fig. 6A, B, Supplementary Fig. 6A). TGF-β-induced p-SMAD2 response was promoted upon IGF2BP3 ectopic expression in MDA-MB-231 cells (Fig. 6C). Moreover, IGF2BP3 upregulated E-cadherin expression while downregulating N-cadherin and Vimentin expression in MCF10A-M2 cells (Fig. 6D). These effects were at least partially reversed by blocking TGF-β signaling with SB505124 (SB; Fig. 6D). Consistently, inhibition of TGF-β signaling with SB significantly mitigated the IGF2BP3-induced increase of migration in MDA-MB-231 cells (Fig. 6E). These findings suggest that IGF2BP3 enhances *TGFBR1* mRNA expression, thereby promoting TGF-β-induced EMT and cell migration.

We then evaluated whether IGF2BP3 is essential for *circTGFBR2(3-6)* to promote TGF-β/SMAD signaling and TGF-β-induced EMT. *IGF2BP3* knockdown using two independent shRNAs reduced the *circTGFBR2(3-6)*-triggered increase in *TGFBR1* mRNA and protein expression, as well as *TGFBR1* mRNA stability, in MDA-MB-231 cells (Fig. 6F–H, Supplementary Fig. 6B, C). However, the interaction between *circTGFBR2(3-6)* and *TGFBR1* mRNA remained unaffected upon *IGF2BP3* knockdown (Supplementary Fig. 6D), suggesting that IGF2BP3 predominantly functions to stabilize *TGFBR1* mRNA, rather than serving as a molecular bridge between *circTGFBR2(3-6)* and *TGFBR1* mRNA. Moreover, we showed that the *circTGFBR2(3-6)*-mediated enhancement of the TGF-β-induced transcriptional response and p-SMAD2 levels was mitigated in the absence of *IGF2BP3* (Fig. 6I, Supplementary Fig. 6E). Furthermore, TGF-β-induced EMT and cell migration, which were potentiated upon *circTGFBR2(3-6)* ectopic expression, were inhibited following *IGF2BP3* depletion in MCF10A-M2 and MDA-MB-231 cells, respectively (Fig. 6J, K). Supporting its pro-tumorigenic role in breast cancer, IGF2BP3 protein expression was higher in basal-type than in luminal-type breast cancer cell lines (Supplementary Fig. 6F). Collectively, these findings highlight IGF2BP3 as a critical effector of *circTGFBR2(3-6)*-induced potentiation of TGF-β/SMAD signaling and TGF-β-induced EMT and cell migration.

### IGF2BP3 binds to and stabilizes m^6^A-modified *TGFBR1* mRNA

**Fig. 7:**
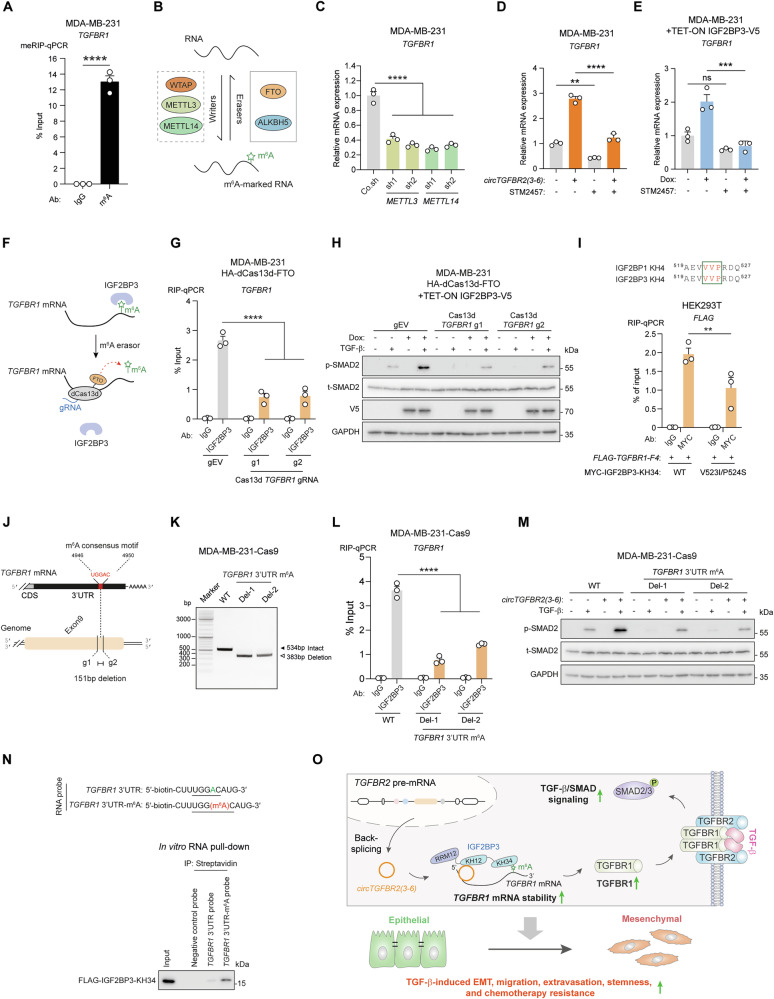
IGF2BP3 binds to and stabilizes m^6^A-modified *TGFBR1* mRNA. **A** m^6^A abundance on *TGFBR1* mRNA in MDA-MB-231 cells, analyzed by methylated RIP (meRIP). RT-qPCR was performed to detect *TGFBR1* in immunoprecipitates. Data are presented as mean ± SEM from three biological replicates, with significance assessed using a two-tailed unpaired Student’s *t*-test. **B** Schematic representation of m^6^A writers (i.e., WTAP, METTL3, and METTL14) and erasers (i.e., FTO and ALKBH5) acting on mRNAs. **C** RT-qPCR analysis of *TGFBR1* mRNA expression in MDA-MB-231 cells upon *METTL3* and *METTL14* depletion. Data are presented as mean ± SEM from three biological replicates, with significance analyzed using one-way ANOVA followed by Dunnett’s multiple comparisons test. RT-qPCR analysis of *TGFBR1* mRNA expression in MDA-MB-231 cells upon ectopic expression of *circTGFBR2(3-6)* (**D**) or IGF2BP3 (using a TET-ON inducible system; **E**), with or without STM2457 treatment. Data are presented as mean ± SEM from three biological replicates, with significance analyzed using one-way ANOVA followed by Dunnett’s multiple comparisons test. **F** Schematic representation of the dCas13d-FTO eraser system for m^6^A (in green) removal from *TGFBR1* mRNA. **G** RIP analysis of the *TGFBR1* mRNA-IGF2BP3 interaction in MDA-MB-231 cells expressing the dCas13d-FTO eraser system and two Cas13d gRNAs targeting *TGFBR1* mRNA. RT-qPCR detected *TGFBR1* in immunoprecipitates. Data are presented as mean ± SEM from three independent experiments, with significance analyzed using two-way ANOVA followed by Šídák’s multiple comparisons test. **H** Effect of IGF2BP3 ectopic expression (using a TET-ON inducible system) and m^6^A removal from *TGFBR1* mRNA on TGF-β-induced p-SMAD2 response in MDA-MB-231 cells. **I** RIP analysis of the interactions between *FLAG-TGFBR1-F4* and either WT or MUT (V523I/P524S) MYC-IGF2BP3-KH34 in HEK293T cells. RT-qPCR detected *FLAG* expression in immunoprecipitates. Data are presented as mean ± SEM from three independent experiments, with significance analyzed using two-way ANOVA followed by Šídák’s multiple comparisons test. **J** Schematic representation of the predicted IGF2BP3-binding consensus motif in the *TGFBR1* 3′UTR, and the gRNA target sites used to generate *TGFBR1* 3′UTR m^6^A deletion. **K** PCR analysis of the *TGFBR1* 3′UTR genomic region in WT MDA-MB-231 cells and two single-cell-derived *TGFBR1* 3′UTR m^6^A deletion clones, visualized by agarose gel electrophoresis. **L** RIP analysis of the *TGFBR1* mRNA-IGF2BP3 interaction in MDA-MB-231 cells with or without *TGFBR1* 3′UTR m^6^A deletion. RT-qPCR detected *TGFBR1* mRNA in immunoprecipitates. Data are presented as mean ± SEM from three independent experiments, with significance analyzed using two-way ANOVA followed by Šídák’s multiple comparisons test. **M** Effect of *circTGFBR2(3-6)* ectopic expression and *TGFBR1* 3′UTR m^6^A deletion on TGF-β-induced p-SMAD2 response in MDA-MB-231 cells. **N** In vitro RNA pull-down analysis of the interaction between the purified FLAG-IGF2BP3-KH34 protein and *TGFBR1* 3′UTR RNA probes with (in red) or without (in green) m^6^A modification. Western blotting with an anti-FLAG antibody was used to detect FLAG-IGF2BP3-KH34 protein expression in the input and immunoprecipitates. **O** Schematic working model. *circTGFBR2(3-6)* is generated by back-splicing of *TGFBR2* exon3-6 in the nucleus and exported to the cytoplasm. There, *circTGFBR2(3-6)* binds to both RNA-binding protein IGF2BP3 and m^6^A-modified *TGFBR1* mRNA to strengthen their interaction, thereby stabilizing *TGFBR1* mRNA. As a consequence, *circTGFBR2(3-6)* promotes TGF-β/SMAD signaling and TGF-β-induced EMT, migration, extravasation, stemness, and chemotherapy resistance in breast cancer cells.

IGF2BP3 has been reported to function as an m^6^A reader that selectively binds to m^6^A-modified mRNAs [19]. Methylated RNA immunoprecipitation (meRIP) followed by RT-qPCR confirmed m^6^A modification on *TGFBR1* mRNA but not on *circTGFBR2(3-6)* (Fig. 7A, Supplementary Fig. 7A). shRNA-mediated depletion of *METTL3* and *METTL14*, which encode key m^6^A methyltransferases (writers) [16], reduced *TGFBR1* mRNA expression, indicating that m^6^A deposition may contribute to *TGFBR1* mRNA stability (Fig. 7B, C, Supplementary Fig. 7B). Moreover, treating MDA-MB-231 cells with STM2457 [57], a highly potent and selective first-in-class catalytic inhibitor of METTL3, significantly decreased *TGFBR1* mRNA expression induced by *circTGFBR2(3-6)* (Fig. 7D) or IGF2BP3 (Fig. 7E) in MDA-MB-231 cells. To further validate these findings, we employed an m^6^A eraser system that fuses catalytically dead Cas13d (dCas13d) to the m^6^A demethylase (eraser) FTO [58], allowing selective removal of m^6^A from *TGFBR1* mRNA (Fig. 7B, F). We selected two gRNAs that efficiently mediated Cas13d-dependent degradation of *TGFBR1* mRNA in MDA-MB-231 cells (Supplementary Fig. 7C). Targeted removal of m^6^A modification from *TGFBR1* mRNA significantly reduced its interaction with IGF2BP3 (Fig. 7G, Supplementary Fig. 7D). As expected, m^6^A eraser-mediated reduction of m^6^A on *TGFBR1* mRNA suppressed IGF2BP3-enhanced *TGFBR1* mRNA expression and TGF-β-induced p-SMAD2 levels in MDA-MB-231 cells (Fig. 7H, Supplementary Fig. 7E). Moreover, mutation of two key residues (V523I/P524S) within an m^6^A-recognition motif of IGF2BP3 [59] reduced the interaction between IGF2BP3-KH34 and the *TGFBR1-F4* fragment (Fig. 7I, Supplementary Fig. 7F). Furthermore, we identified a UGGAC RNA consensus motif, which contains the GGAC m^6^A core motif preferentially recognized by IGF2BPs [19], at positions of 4946-4950 in the *TGFBR1* 3′UTR (Fig. 7J). We employed a CRISPR-Cas9-based strategy to delete a 151-bp genomic fragment containing the sequence encoding the UGGAC RNA consensus motif in MDA-MB-231 cells, using paired gRNAs (Fig. 7J). The interaction between IGF2BP3 protein and *TGFBR1* mRNA was diminished in two independent MDA-MB-231 single clones lacking the UGGAC RNA consensus motif (Fig. 7K, L). Consistently, deletion of this motif reduced the *circTGFBR2(3-6)*-directed increase in *TGFBR1* mRNA expression and TGF-β-induced p-SMAD2 response (Fig. 7M, Supplementary Fig. 7G). Furthermore, in vitro RNA pull-down assays demonstrated that an RNA probe containing this motif and its flanking sequences interacted with the purified IGF2BP3-KH34 protein, whereas a negative control probe did not (Fig. 7N, Supplementary Fig. 7H). Notably, *N*^6^-methylation of the adenosine in the GGAC RNA consensus motif significantly enhanced IGF2BP3 binding (Fig. 7N). Taken together, our results suggest that IGF2BP3 directly binds to m^6^A-modified *TGFBR1* mRNA to promote its stability and thereby potentiates TGF-β/SMAD signaling.

## Discussion

circRNAs are emerging as a new class of modulators of TGF-β signaling in cancer [60, 61]. In this study, we identify *circTGFBR2(3-6)* as a potent enhancer of TGF-β/SMAD signaling and TGF-β-induced EMT, migration, extravasation, stemness, and chemotherapy resistance in breast cancer cells (Fig. 7O). *circTGFBR2(3-6)* binds to both RNA-binding protein IGF2BP3 and *TGFBR1* mRNA to strengthen their interaction, and thereby stabilizes *TGFBR1* mRNA in an m^6^A-dependent manner (Fig. 7O). We predicted two putative inverted *Alu cis*-acting elements in the exon-flanking regions of *TGFBR2* exon3-6, which may contribute to its circularization. Depleting a *TGFBR2* intron2 fragment containing the *Alu* element was sufficient to reduce *circTGFBR2(3-6)* expression. The back-splicing of circRNAs can be facilitated by both *cis*-acting elements within flanking introns [39, 62] and by *trans*-acting RNA-binding proteins that bind *cis*-regulatory elements on pre-mRNAs [63, 64]. Quaking (QKI) has been shown to bind to exon-flanking sequences of circRNAs to facilitate their circularization during TGF-β-induced EMT [63]. A recent study demonstrated that TGF-β upregulates *circITGB6(11,12)* expression at the transcriptional level to promote TGF-β-induced EMT in multiple cell lines [65]. However, TGF-β did not affect *circTGFBR2(3-6)* expression in our study. Further investigation may focus on identifying the RNA-binding proteins that regulate *circTGFBR2(3-6)* biogenesis and elucidating the upstream pathways that may cross-talk with TGF-β receptor signaling to enhance EMT.

We have eliminated several potential mechanisms by which *circTGFBR2(3-6)* promotes TGF-β/SMAD signaling. Small peptides can be translated from circRNAs to elicit their functions [32, 33]. A recent study in *Drosophila* showed that *TGFBR1* pre-mRNA-derived *circbabo(5,6,7,8S)* encodes the protein circbabo, which disrupts the assembly of the TGFBR1/2 heterodimer complex [66]. Our results showed that mutating all ATG codons in *circTGFBR2(3-6)* did not affect its role in promoting TGF-β-induced p-SMAD2 response, validating the notion that *circTGFBR2(3-6)* serves as a non-coding circRNA to potentiate TGF-β/SMAD signaling. Another well-established mechanism of circRNA function is acting as sponges for miRNAs to prevent their binding to target mRNAs [30, 31]. In particular, two other circRNAs produced from *TGFBR2* pre-mRNA—*circTGFBR2(4)* and *circTGFBR2(2,3)*—which have distinct exon compositions from *circTGFBR2(3-6)*, have been reported to sponge miRNAs in aortic dissection [67], nasopharyngeal carcinoma [68], and hepatocellular carcinoma [69]. However, we ruled out this possibility by depleting *AGO2* to disrupt the function of RISC [51]. Although *circTGFBR2(3-6)* is expressed at lower levels than *TGFBR1* mRNA, it can still exert a significant regulatory effect on *TGFBR1* mRNA stability. This may be attributed to its inherent stability, conferred by its covalently closed loop structure, which renders it more resistant to exonuclease-mediated degradation compared to linear RNAs [25, 26], including *TGFBR1* mRNA. As a result, *circTGFBR2(3-6)* may accumulate over time and sustain functionally relevant concentrations within the IGF2BP3/*circTGFBR2(3-6)*/*TGFBR1* mRNA ternary complex for *TGFBR1* mRNA stabilization.

We employed mass spectrometry-based interactome analysis to identify protein partners of *circTGFBR2(3-6)* and selected IGF2BP3 for investigation, given its established role in RNA stabilization [19, 53]. Our prior observation indicated that *TGFBR1* mRNA half-life was upregulated by *circTGFBR2(3-6)* (Fig. 4E). However, we cannot fully eliminate the potential involvement of other *circTGFBR2(3-6)*-interacting proteins in mediating its biological effects. circRNAs have been reported to interact with IGF2BP3, thereby preventing it from degradation [70, 71], changing its protein conformation [65], competing its binding to target mRNAs [72], or forming a tertiary complex with target mRNAs [73]. For example, TGF-β-induced *circITGB6(11,12)* binds to IGF2BP3 and enhances its interaction with *podoplanin* (*PDPN*) mRNA, thereby promoting TGF-β-induced EMT [65]. *circTGFBR2(3-6)* depletion diminished the interaction between IGF2BP3 protein and *TGFBR1* mRNA, underscoring its role as a scaffold in reinforcing this protein-mRNA interaction.

Our in vitro RNA pull-down experiments demonstrated a direct interaction between IGF2BP3-KH12 and two CA-N_15-25_-CGGCA RNA consensus motifs within *circTGFBR2(3-6)*. Of note, this RNA consensus sequence was not detected in *TGFBR1* mRNA, which may explain the lack of association between IGF2BP3-KH12 and *TGFBR1* mRNA. In contrast, in vitro RNA pull-down assays demonstrated that IGF2BP3-KH34 di-domain directly binds to the m^6^A consensus sequence in the 3′UTR of *TGFBR1* mRNA, consistent with previous studies demonstrating that IGF2BP3-KH34 mediates the recognition and binding of m^6^A-modified mRNAs [19]. Importantly, RIP-qPCR data showed that either removal of m^6^A using the dCas13d-FTO eraser system or disruption of the genomic region encoding the m^6^A consensus site in the *TGFBR1* 3′UTR impaired IGF2BP3 binding to *TGFBR1* mRNA, highlighting the critical role of m^6^A in this interaction. Consistently, m^6^A was not detected in *circTGFBR2(3-6)*, which may explain why IGF2BP3-KH34 interacts with *TGFBR1* mRNA but not with *circTGFBR2(3-6)*. A hydrophobic cradle (^522^VVP^524^) within the KH4 domain of IGF2BP1, which is highly conserved across IGF2BP family members, has been shown to recognize m^6^A modifications in a manner dependent on the cellular concentration of available IGF2BP1 and the GGAC sequence context [59]. Consistent with this, mutation of two key residues within this hydrophobic cradle reduced the interaction between IGF2BP3-KH34 and *TGFBR1* mRNA. However, we cannot exclude potential contributions of other motifs within IGF2BP3 to its interaction with *TGFBR1* mRNA.

We showed that ectopic expression of shRNA-resistant *circTGFBR2(3-6)* rescued the inhibition of TGF-β/SMAD signaling resulting from endogenous *circTGFBR2(3-6)* knockdown. This indicates that introducing multiple mutations into the BSJ of *circTGFBR2(3-6)* does not impair its effect on TGF-β/SMAD signaling. Notably, TGF-β-induced p-SMAD2 response remained unchanged upon ectopic expression of linear *TGFBR2 exon3-6*. Despite sharing common RNA-binding motifs, certain circRNAs and their linear counterparts exhibit diverse binding affinities for RNA-binding proteins [74–76]. Moreover, the *circTGFBR2(3-6)* BSJ mutant retained its ability to bind *TGFBR1* mRNA as efficiently as the wild-type *circTGFBR2(3-6)*. Therefore, the circular conformation of *circTGFBR2(3-6)*, rather than its unique BSJ, likely determines the specificity to interact with *TGFBR1* mRNA and to promote TGF-β/SMAD signaling.

Our results demonstrated that blocking TGF-β signaling mitigated the enhancement of EMT and migration mediated by *circTGFBR2(3-6)* and its effector IGF2BP3 in breast cancer cells. We found that IGF2BP3 protein expression was increased in the basal-type breast cancer cell lines compared to luminal cell lines, suggesting its pro-tumorigenic function. IGF2BP3 promotes EMT and migration across different types of cancer cells through various mechanisms [77–79]. Given the pivotal role of TGF-β signaling in EMT induction, it is likely that the EMT-promoting effects of IGF2BP3 observed in these studies are, at least partially, due to its role in potentiating TGF-β signaling via *circTGFBR2(3-6)*. We found that IGF2BP3-enhanced TGF-β/SMAD signaling by binding to and increasing *TGFBR1* mRNA stability. This is supported by the previous RNA immunoprecipitation sequencing (RIP-seq) [19, 80, 81] and photoactivatable ribonucleoside-enhanced crosslinking and immunoprecipitation sequencing (PAR-CLIP-seq) [82] analyses, which identified *TGFBR1* mRNA as a high-confidence IGF2BP3-binding target in multiple cell lines. Additionally, *TGFBR1* is among the significantly downregulated genes upon *IGF2BP3* depletion in pancreatic cancer cells [80], which aligns with our results demonstrating that IGF2BP3 promotes *TGFBR1* mRNA expression in breast cancer cells. IGF2BP3 acts as an m^6^A reader to recognize and bind to m^6^A-marked mRNAs to enhance their stability and translation [19]. We observed that IGF2BP3 ectopic expression upregulated TGFBR1 protein expression to a similar extent as the upregulation of *TGFBR1* mRNA, suggesting that the protein increase is primarily driven by mRNA stabilization. However, whether IGF2BP3 also facilitates *TGFBR1* mRNA translation, in addition to enhancing its stability, requires further investigation.

Our work unravels a novel mechanism by which *TGFBR2* pre-mRNA-derived *circTGFBR2(3-6)* enhances *TGFBR1* mRNA stability, adding an additional layer of regulation to TGF-β receptor signaling. Given the tissue- and cell-specific expression of circRNAs, future investigation could profile *circTGFBR2(3-6)* in breast cancer patients and evaluate strategies to inhibit its function to suppress overactive TGF-β signaling in cancer cells. Furthermore, since *circTGFBR2(3-6)* promotes TGF-β/SMAD signaling in an m^6^A-modified *TGFBR1* mRNA-dependent manner, it will be interesting to investigate whether targeting METTL3 with STM2457 [57] or disrupting IGF2BP3 binding to m^6^A-modified RNA using the small-molecule inhibitor I3IN-002 [83] could inhibit TGF-β-driven EMT and cancer progression.

## Materials and methods

### Cell culture and reagents

HEK293T (CRL-1573), A549 (CRM-CCL-185), MDA-MB-231 (CRM-HTB-26), MDA-MB-436 (HTB-130), HCC38 (CRL-2314), and BT549 (HTB-122) cells were purchased from the American Type Culture Collection (ATCC). SUM149PT and HCC1806 cells were obtained from Dr. Sylvia Le Dévédec (Leiden Academic Center for Drug Research, Leiden, the Netherlands). HACAT cells were obtained from Dr. N. E. Fusenig (German Cancer Research Center, Heidelberg, Germany) and have been previously described [84]. Bone metastatic MDA-MB-231 (MDA-MB-231-BM) cells were obtained as previously described [85]. All cell lines were cultured in Dulbecco’s modified Eagle medium (DMEM; 41965062; Thermo Fisher Scientific, Paisley, UK) supplemented with 10% fetal bovine serum (FBS; S1810-500; Biowest, Nuaillé, France) and 100 U/mL penicillin/streptomycin (15140122; Gibco, Bleiswijk, the Netherlands). Details of the other cell lines in the panel of 20 breast cancer cell lines (Fig. 3A) have been previously described [86]. MCF10A-M2 cells, kindly provided by Dr. Fred Miller (Barbara Ann Karmanos Cancer Institute, Detroit, USA), were cultured in DMEM/F12 (GlutaMAX™ Supplement; 31331; Thermo Fisher Scientific, Paisley, UK) containing 5% horse serum (26050088; Thermo Fisher Scientific, Paisley, UK), 0.1 μg/mL cholera toxin (C8052; Sigma‒Aldrich, Darmstadt, Germany), 0.02 μg/mL epidermal growth factor (EGF; 01-107, Sigma‒Aldrich, Darmstadt, Germany), 0.5 μg/mL hydrocortisone (H0135; Sigma‒Aldrich, St. Louis, MO, USA), 10 μg/mL insulin (I6634, Sigma‒Aldrich, Darmstadt, Germany), and 100 U/mL penicillin/streptomycin. All cell lines were maintained in a 5% CO_2_, 37 °C humidified incubator, authenticated by short tandem repeat (STR) profiling, and tested monthly for mycoplasma contamination. Recombinant TGF-β3 is a kind gift provided by Dr. Andrew Hinck (University of Pittsburgh, USA). The following reagents were used in cell culture experiments: Actinomycin D (ActD, 1 μM; A9415; Sigma‒Aldrich, Darmstadt, Germany), doxorubicin (5-day treatment; D5220; Sigma‒Aldrich, Darmstadt, Germany), paclitaxel (5-day treatment; T7191; Sigma‒Aldrich, Darmstadt, Germany), doxycycline (100 ng/mL for 2 days; D9891-1G, Sigma‒Aldrich, Darmstadt, Germany), and STM2457 (10 μM; S9870; Selleckchem, Cologne, Germany).

### circRNA screen

MDA-MB-231 cells (4 × 10^4^) with stable expression of the CAGA_12_-dynGFP reporter [37] were seeded into 96-well plates and transduced with lentivirus carrying shRNAs against *TGFBR1* or *TGFBR2* pre-mRNA-derived circRNAs for 16 h. shRNAs targeting linear *TGFBR1* or *TGFBR2* mRNA were taken along as controls. After transduction, cells were serum-starved for 16 h and stimulated with TGF-β (0.5 ng/mL). The TGF-β-induced transcriptional response was monitored using the IncuCyte live cell imaging system (Essen BioScience, Newark, UK). Relative reporter activity was quantified as the total integrated GFP intensity, normalized to cell confluence. The results at 21 h post-TGF-β stimulation are presented. The experiment was performed twice, and representative results are shown.

### In situ hybridization

The BaseScope™ Reagent Kit v2-RED (323900; Advanced Cell Diagnostics, Newark, CA, USA) and a probe targeting the *circTGFBR2(3-6)* BSJ sequence (1321951-C1; Advanced Cell Diagnostics, Newark, CA, USA) were utilized to assess the subcellular localization of *circTGFBR2(3-6)* in MDA-MB-231 and A549 cells. Imaging was performed using a DMi8 inverted fluorescence microscope (Leica). Representative results from two independent experiments are presented.

### Plasmid construction

*TGFBR2 exon3-6* was amplified using PCR from MDA-MB-231 cell-derived cDNA and inserted into pf-CAG-mc2-internal ribosomal entry site (IRES)-blasticidin (blast) [41] (a gift from Simon Conn & Brett Stringer, Addgene; 206235) and pCDH-elongation factor (EF)1α-MCS-polyA-Blast (System Biosciences, CA, USA) for the expression *circTGFBR2(3-6)* and linear *TGFBR2 exon3-6*, respectively. The *circTGFBR2(3-6)* ATG mutant (all ATGs were mutated to ATTs; synthesized as a mini-gene by Integrated DNA Technologies, Leuven, Belgium) and the *circTGFBR2(3-6)* shRNA-resistant mutant were ligated into the abovementioned pf-CAG-mc2-IRES-blast construct. *circTGFBR2(3-6)* shRNA constructs were generated by oligo re-annealing and ligation into the pLKO.1-U6-puromycin (PURO) construct (Sigma‒Aldrich). For CRISPR/Cas9-mediated genomic deletion and Cas13d-mediated *TGFBR1* mRNA targeting, gRNAs were inserted into the lentiviral vectors pLKO.1-U6-PURO-AA19 [87] (kindly provided by Dr. Manuel A.F.V. Gonçalves, LUMC) and pLKO.1.CasRx-gRNA-PURO (modified from pLKO.1-U6-PURO), respectively. The TET-ON inducible construct for IGF2BP3 ectopic expression was generated using Gateway cloning into the pLIX-403 vector (a gift from David Root, Addgene; 41395). IGF2BP3 and *TGFBR1* mRNA truncation mutants were cloned into the pcDNA3.MYC and pcDNA3.FLAG vectors, respectively. Expression constructs of MYC-IGF2BP3-KH34 (V523I/P524S) and *FLAG-TGFBR1-F1-MUT* were synthesized as mini-genes (Integrated DNA Technologies, Leuven, Belgium) and subcloned into their respective expression vectors. All plasmids were verified by Sanger sequencing, and the primers used for plasmid construction are listed in Supplementary Table 2.

### Lentiviral transduction

Lentivirus production and transduction were performed as previously described [88]. shRNA constructs from Sigma‒Aldrich were used for the knockdown of the following targets: *TGFBR1* (TRCN0000039773), *TGFBR2* (CDS: TRCN0000040012, 3′UTR: TRCN0000197031 (sh1) and TRCN0000194992 (sh2)), *IGF2BP3* (TRCN0000286268 (sh1) and TRCN0000293596 (sh2)), *METTL3* (TRCN0000034715 (sh1) and TRCN0000034717 (sh2)), and *METTL14* (TRCN0000015933 (sh1) and TRCN0000015936 (sh2)). plentiCRISPR.v2-PURO [89] (a gift from Dr. Brett Stringer, Addgene; 98290), pXR001-EF1a-CasRx-2A-EGFP [90] (a gift from Dr. Patrick Hsu, Addgene; 109049), and plenti-EF1α-dCasRx-FTO-HA-T2A-BSD [58] (a gift from Dr. Wenbo Li, Addgene; 177120) were used for the construction of MDA-MB-231 stable cell lines.

### siRNA transfection

MDA-MB-231 cells (1.2 × 10^5^) were seeded in 12-well plates and transfected with Lipofectamine 3000 (L3000015; Thermo Fisher Scientific, Vilnius, Lithuania) with 25 nM of either non-targeting siRNA (si-NT; Horizon Cambridge, UK), si-*circTGFBR2(3-6)* (GUCGUUAUUAACUCCCACUGCAUU; Horizon, Cambridge, UK), or si-*AGO2* (L-004639-00-0005; Horizon, Cambridge, UK). The medium was replaced after 6 h. A second identical transfection was performed 48 h later, and samples were collected 48 h post-transfection.

### RT‒qPCR

To detect the expression of TGF-β target genes, MDA-MB-231 cells were treated with TGF-β (1 ng/ml) or a vehicle control for 8 h. Total RNA was isolated using the NucleoSpin RNA kit (740955; Macherey Nagel, Duren, Germany), and reverse transcription was performed with the RevertAid RT Reverse Transcription Kit (K1691; Thermo Fisher Scientific, Vilnius, Lithuania). The indicated genes were amplified using cDNA and specific primer pairs (listed in Supplementary Table 3). Gene expression was quantified using the CFX Connect Real-Time PCR Detection System (Bio-Rad, Hercules, CA, USA), with *GAPDH* as the reference gene for normalization by the 2^−ΔΔCt^ method. All experiments were performed at least three times, and representative results are shown.

### RNase R treatment

MDA-MB-231 cells were treated with RNase R (1 U/μl; ab286929; Abcam, Cambridge, UK) for 2 h at 37 °C. RNA samples were collected for RT-qPCR analysis.

### Enriched circRNA pool (ECP)

Full-length circRNAs were enriched from MDA-MB-231 cells using the protocol established by L. Hou et al. [91]. In brief, prior to poly(A) tailing, 60 μg of total RNA was heated at 70 °C for 5 min, followed by 5-min incubation on ice. Subsequently, 17.5 μl of 10 mM Adenosine 5′-Triphosphate (ATP) (P0756S; NEB, Ipswich, MA, USA), 7 μl of 5 U/μl E. coli Poly(A) Polymerase (M0276; NEB, Ipswich, MA, USA), 3.5 μl of 40 U/μl RiboLock RNase Inhibitor (EO0381; Thermo Fisher Scientific, Vilnius, Lithuania) and 17.5 μl of 10× E. coli Poly(A) Polymerase reaction buffer were directly added to the sample, with a final volume of 175 μl. The resulting mixture was incubated at 37 °C for 30 min and then purified with RNA Clean & ConcentratorTM-5 (R1016; ZYMO RESEARCH, Orange, CA, USA). Next, the mixture was subjected to 1 U/μl RNase R treatment for 1 h at 37 °C, after which the poly(A)^+^ RNA fraction was removed using Dynabeads™ Oligo(dT)_25_ (61002; Thermo Fisher Scientific, Vilnius, Lithuania) following the manufacturer’s instructions. Residual remaining ribosome RNA (rRNA) in the obtained poly(A)^-^ RNA supernatant was subsequently depleted with Ribo-Zero Plus rRNA Depletion Kit (20037135; Illumina, San Diego, CA, USA) according to the manufacturer’s protocol. Finally, the enriched circRNA pool was used as a template for reverse transcription, and RT-qPCR was performed to validate the enrichment of circRNAs by assessing the expression of selected rRNAs, linear transcripts, and circRNAs.

### Western blotting

To detect EMT marker expression, MCF10A-M2 cells were treated with TGF-β (2.5 ng/ml) or vehicle control for 3 days in DMEM medium. To check TGF-β-induced p-SMAD2 response, cells were treated with TGF-β (1 ng/ml) or vehicle control for 1 h. Western blotting was performed as previously described [88]. The primary antibodies used are listed in Supplementary Table 4. All experiments were performed at least three times, and representative results are shown. Uncropped blots are shown in a single supplementary file.

### Transcriptional reporter assay

MDA-MB-231 cells with stable expression of the CAGA_12_-dynGFP reporter [37] were used to monitor the TGF-β-induced transcriptional response using the IncuCyte live cell imaging system (Essen BioScience, Newark, UK). Cells were serum-starved for 16 h and then stimulated with TGF-β (0.5 ng/mL) or vehicle control. Relative reporter activity was quantified as total green integrated GFP intensity normalized to cell confluence. All experiments were performed three times, and representative results are shown.

### F-actin staining

A549 cells were treated with TGF-β (5 ng/mL in Fig. 3F and 1  ng/mL in Fig. 3G), SB505124 (SB; 1 μM; 3263; Tocris, Abingdon, UK), or vehicle control for 48 h. Cells were then stained with Phalloidin conjugated to Alexa Fluor 488 (1:500 dilution; A12379; Thermo Fisher Scientific, Bleiswijk, the Netherlands), as previously described [92]. Images were acquired with a Leica SP8 confocal microscope (Leica Microsystems). Experiments were performed twice, and representative results are shown.

### MTS tetrazolium cell proliferation and viability assay

MTS assays were performed to quantify cell proliferation and viability, following the manufacturer’s instructions (G3581; Promega, Madison, WI, USA). Cells were seeded at a density of 1 × 10^3^ cells in wells of 96-well plates (Corning). Cells were stimulated with TGF-β (5 ng/mL) or vehicle control for 5 days. The absorbance of the samples was measured at 490 nm with a luminometer.

### Transwell migration assay

Cells were seeded in an IncuCyte Clearview 96-well plate (4582; Essen BioScience, Newark, UK), and chemotactic cell migration was monitored using the IncuCyte live cell imaging system (Essen BioScience, Newark, UK) as previously described [92]. Cells were treated with TGF-β (5 ng/mL; added to both top and bottom chambers) or vehicle control during the assays. Cells in both the top and bottom chambers were imaged and quantified using the IncuCyte system. Experiments were performed twice, and representative results are shown.

### Subcellular fractionation

Cytoplasmic and nuclear fractions were collected from MDA-MB-231 or MCF10A-M2 cells as previously described [88]. Experiments were performed three times, and representative results are shown.

### RNA immunoprecipitation (RIP)

RIP was performed using the Magna RIP™ RNA-Binding Protein Immunoprecipitation Kit (17-700; Merck Millipore, Rockford, IL, USA) as previously described [93]. Briefly, anti-IGF2BP3 antibody (ab177477; Abcam, Cambridge, UK), anti-MYC antibody (M4439; Sigma‒Aldrich, Darmstadt, Germany), or a normal IgG control was incubated with cell lysates for 16 h at 4 °C. RNA was extracted from the beads, and RT‒qPCR was performed as described above. Experiments were performed three times, and representative results are shown.

### Methylated RIP (meRIP)

10 μg of RNA extracted from MDA-MB-231 cells was diluted with 1250 μL RIP buffer (25 mM Tris-HCl, pH 7.5, 150 mM KCl, 5 mM EDTA, 0.5 mM DTT, and 0.5% NP-40). 100 μL of diluted RNA was preserved as 10% input. 5 μg of m^6^A antibody (ab151230; Abcam, Cambridge, UK) or a normal IgG control was added to 500 μL of diluted RNA and incubated with rotation at 4 °C for 16 h. The RNA-antibody complex was then captured by adding 30 μL of Protein A Sepharose beads (17-0963-03; GE Healthcare, Uppsala, Sweden), followed by an additional incubation with rotation for 3 h at 4 °C. After incubation, the beads were washed three times with RIP buffer. RNA was extracted from both the beads and the input sample, as described above.

### Genomic deletion by CRISPR-Cas9

MDA-MB-231 cells stably expressing Cas9 were transduced with combinations of paired gRNA expression constructs by lentivirus infection. Genomic DNA was isolated using the DNeasy Blood & Tissue kit (69504; Qiagen, Hilden, Germany). The genomic region was characterized by PCR-based genotyping with LongAmp® Taq DNA Polymerase (M0323S; NEB, Ipswich, MA, USA). MDA-MB-231-Cas9 cells transduced with an empty gRNA expression vector served as control (WT).

### RNA pull-down and mass spectrometry

Four 15-cm dishes of MDA-MB-231 cells were collected by centrifugation at 500 × *g* for 5 min at 4 °C. The cell pellets were washed twice with cold phosphate buffered saline (PBS) and resuspended in 3 mL of lysis buffer (25 mM HEPES, pH 7.4; 150 mM NaCl; 10% Glycerol; 5 mM EDTA; and 1% TritonX-100) supplemented with freshly added protease inhibitor cocktail (11836153001; Roche, Mannheim, Germany) and Ribolock RNase inhibitor (100 U/mL; EO0382; Thermo Fisher Scientific, Bleiswijk, the Netherlands). Cells were lysed on ice for 20 min, and the supernatant was collected by centrifugation at 12,000 × *g* for 15 min at 4 °C. A portion of the cell lysate (5% and 1%) was set aside as input for RT-qPCR and western blotting, respectively. The remaining lysate was divided into two parts and pre-cleared by rotation with 30 μl Dynabeads M-270 Streptavidin (65305; Invitrogen, Rockford, IL, USA) and 15 μl yeast tRNA (AM7119; Thermo Fisher Scientific, Bleiswijk, the Netherlands) for 1 h at 4 °C. Pre-cleared lysates were then incubated with 5 μl of 3′-triethyleneglycol (TEG) biotinylated probes (100 μM; Integrated DNA Technologies, Leuven, Belgium) targeting either *LacZ* (CCAGTGAATCCGTAATCATG), *circTGFBR2(3-6)* (TGCAGTGGGAGTTAATAACGAC), or *FLAG* (GAATTCGCCCTTGTCATCATCGTCCTTGTAGTCCATGGC) under rotation for 16 h at 4 °C. Streptavidin Dynabeads were pre-blocked with Bovine Serum Albumin (BSA; A9647-100G; Sigma‒Aldrich, Darmstadt, Germany) and yeast tRNA to minimize non-specific binding before adding to the cell lysates. The beads were incubated with rotation for 3 h at 4 °C to capture the biotinylated probes. Following incubation, the beads were washed three times with lysis buffer. For western blotting, the beads were boiled in sample buffer for 5 min, and proteins of interest were analyzed. For RT-qPCR, RNA was extracted from the beads as described above. For mass spectrometry analysis, the beads were washed with 50 mM ammonium bicarbonate, resuspended in 250 μl of 50 mM ammonium bicarbonate, and incubated with 250 ng of trypsin (V5280; Promega, Madison, WI, USA) for 16 h at 37 °C. Finally, the beads were separated using a prewashed 0.4-μm filter (UFC30HV00; Millipore, Rockford, IL, USA). Peptides were dissolved in water/formic acid (100/0.1 v/v) and analyzed by online C18 nano high-performance liquid chromatography (HPLC) tandem mass spectrometry (MS/MS) with a system consisting of an Ultimate3000nano gradient HPLC system (Thermo, Bremen, Germany), and an Exploris480 mass spectrometer (Thermo). Fractions were injected onto a cartridge precolumn (300 μm × 5 mm, C18 PepMap, 5 μm, 100 A), and eluted via a homemade analytical nano-HPLC column (30 cm × 75 μm; Reprosil-Pur C18-AQ 1.9 μm, 120 A (Dr. Maisch, Ammerbuch, Germany)). The gradient was run from 2% to 36% solvent B (20/80/0.1 water/acetonitrile/formic acid (FA) v/v) in 120 min at 250 nl/min. The nano-HPLC column was drawn to a tip of ∼10 μm and acted as the electrospray needle of the MS source. The mass spectrometer was operated in data-dependent Top 20 MS/MS mode, with a higher energy collision dissociation (HCD) at 30% and recording of the MS2 spectrum in the orbitrap, with a quadrupole isolation width of 1.2 Da. In the master scan (MS1), the resolution was 120,000, the scan range 300–1500, at standard AGC target and a maximum fill time of 50 ms. A lock mass correction on the background ion m/z = 445.12003 was used. Precursors were dynamically excluded after n = 1 with an exclusion duration of 45 s, and with a precursor range of 20 parts per million (ppm). Included charge states were 1–6. For MS2, the first mass was set to 120 Da, and the MS2 scan resolution was 30,000 at an AGC target of standard at a maximum fill time of 60 ms. In a post-analysis process, raw data were first converted to peak lists using Proteome Discoverer version 2.5 (Thermo Scientific), and then submitted to the Uniprot human minimal database (20596 entries), using Mascot v. 2.2.07 (www.matrixscience.com) for protein identification. Mascot searches were done with 10 ppm and 0.02 Da deviation for precursor and fragment mass, respectively, and trypsin was specified as the enzyme. Methionine oxidation was set as a variable modification. Carbamidomethyl was set as a fixed modification on cysteines. The false discovery rate was set to < 1%. We set coverage >35% and number of unique peptides >10 as a cut-off to enrich *circTGFBR2(3-6)*-interacting proteins.

### Protein expression and purification

FLAG-tagged IGF2BP3-KH12 and IGF2BP3-KH34 were expressed separately in HEK293T cells. Cells from five 15-cm dishes were harvested and lysed in 10 mL lysis buffer (25 mM HEPES, pH 7.4; 150 mM NaCl; 10% Glycerol; 5 mM EDTA; and 1% TritonX-100) on ice for 20 min. The lysate was clarified by centrifugation at 12,000 × *g* for 15 min at 4 °C, and the supernatant was collected. 80 μl of anti-FLAG M2 resin (A2220; Sigma–Aldrich, Darmstadt, Germany) was added and incubated for 30 min at 4 °C to affinity purify FLAG-tagged proteins. After three washes with lysis buffer, the bound proteins were eluted by rotating the beads with FLAG peptide (F3290; Merck Millipore, Rockford, IL, USA) at a final concentration of 100 μg/mL for 1 h at 4 °C. Protein purity was assessed by SDS–PAGE followed by Coomassie staining.

### In vitro RNA pull-down

40 nM 5′-triethylene glycol (TEG)-linked biotinylated RNA probes (Integrated DNA Technologies, Leuven, Belgium) were incubated with 50 nM purified FLAG-IGF2BP3 truncation mutant proteins for 16 h at 4 °C. Subsequently, 30 μl of pre-blocked Dynabeads M-270 Streptavidin (65305; Invitrogen, Rockford, IL, USA) were added and incubated with rotation for 3 h at 4 °C. After incubation, the beads were washed three times with low-salt lysis buffer (25 mM HEPES, pH 7.4; 150 mM NaCl; 10% Glycerol; 5 mM EDTA; and 1% TritonX-100) and twice with high-salt lysis buffer (25 mM HEPES, pH 7.4; 300 mM NaCl; 10% Glycerol; 5 mM EDTA; and 1% TritonX-100). Bound proteins were eluted by boiling the beads for 5 min and analyzed by western blotting. Experiments were performed three times, and representative results are shown. The sequences of RNA probes are listed in Supplementary Table 5.

### RNA-seq-based transcriptional profiling and GSEA

To screen for mRNAs affected by *circTGFBR2(3-6)*, the DNBSeq platform (BGI, Poland) was used to perform RNA-seq in MDA-MB-231 cells upon *circTGFBR2(3-6)* ectopic expression. RNA-seq reads were processed using the opensource BIOWDL RNA-seq pipeline v5.0.0 (https://eur03.safelinks.protection.outlook.com/?url=https%3A%2F%2Fzenodo.org%2Frecord%2F5109461%23.Ya2yLFPMJhE&data=05%7C02%7Cc.fan%40lumc.nl%7C745ef3b26cca4179ad3108dcb5216766%7Cc4048c4fdd544cbd80495457aacd2fb8%7C0%7C0%7C638584402282389605%7CUnknown%7CTWFpbGZsb3d8eyJWIjoiMC4wLjAwMDAiLCJQIjoiV2luMzIiLCJBTiI6Ik1haWwiLCJXVCI6Mn0%3D%7C0%7C%7C%7C&sdata=f%2FKAhFLm%2BzgqqZEAhf0O1ns2ygC5sFlRJAgCXN5qgPs%3D&reserved=0) developed at the LUMC. This pipeline performs FASTQ preprocessing (including quality control, quality trimming, and adapter clipping), alignment, read quantification, and optionally transcript assembly. FastQC (v0.11.9) was used for checking raw read quality control (QC). Adapter clipping was performed using Cutadapt (v2.10) with the default settings. RNA-seq reads’ alignment was performed using STAR (v2.7.5a) on human reference genome GRCh38. The gene read quantification was performed using HTSeq-count (v0.12.4) with the Ensembl gene annotation version 114. The TGF-β gene signature [94] was used to perform GSEA with the GSEA software [95].

### Embryonic zebrafish extravasation assay

The experiments were conducted in a licensed establishment for the breeding and use of experimental animals (at Leiden University), and we adhered to internal regulations and guidelines. Advice from the animal welfare body was followed to minimize suffering within the facility. The zebrafish assays described do not fall under the definition of an animal experiment according to the Experiments on Animals Act (Wod, effective 2014), the applicable legislation in the Netherlands, in compliance with European guidelines (EU directive 2010/63/EU) on the protection of animals used for scientific purposes. Since non-self-feeding larvae were used, no specific license was required for these assays on zebrafish larvae (<5 days post-fertilization). Zebrafish isolated from eggs were mixed in one plate containing egg water to randomize them before the injection of fluorescently labeled cancer cells with genetic manipulation. For the number of zebrafish used in the study, sample sizes were determined according to experience with previous experiments [46, 88, 93, 96]. MDA-MB-231 cells labeled with mCherry were injected into the duct of Cuvier of transgenic zebrafish embryos (*fli*; enhanced green fluorescence protein (EGFP)), as previously described [46]. The experiments were performed in a non-blinded manner. An inverted SP5 STED confocal microscope (Leica) was used to visualize the injected cancer cells and zebrafish embryos.

### Statistical analysis

Statistical analysis was performed using GraphPad Prism 10.2.3. *P* < 0.05 was considered statistically significant. All measurements in this study were taken from distinct samples. No samples or animals were excluded from the analysis. The variance is similar between the groups that are being statistically compared. All experiments were performed in a non-blinded manner, because the experimental design was complicated and blinding feasibility was poor. *0.01  <  P  <  0.05; **0.001  <  P  <  0.01; ***0.0001  <  P  <  0.001; ****P  <  0.0001; ns not significant.

## Supplementary information

Supplementary figures and tables

Uncropped blots

circTGFBR2(3-6) predicted 3D structure

## Data Availability

The mass spectrometry proteomics data have been deposited to the ProteomeXchange Consortium (www.proteomexchange.org) by the PRIDE partner repository [97] with the dataset identifier PXD061218. The RNA-seq data generated in this study have been deposited in the GEO database under accession code GSE303132. All other data needed to evaluate the conclusions in the paper are present in the paper or the Supplementary Materials.
