# Supplementary material for: circTGFBR2(3-6) acts as an assembly platform for RNA-binding protein IGF2BP3 and TGFBR1 mRNA to enhance breast cancer cell plasticity
Source: Cell Death Differ. 2025 Oct 27;33(4):779–97. doi: 10.1038/s41418-025-01597-2 (PMC13077047; doi:10.1038/s41418-025-01597-2)
Supplement: Supplementary file 1 — Supplementary figures and tables [file 41418_2025_1597_MOESM1_ESM.docx]

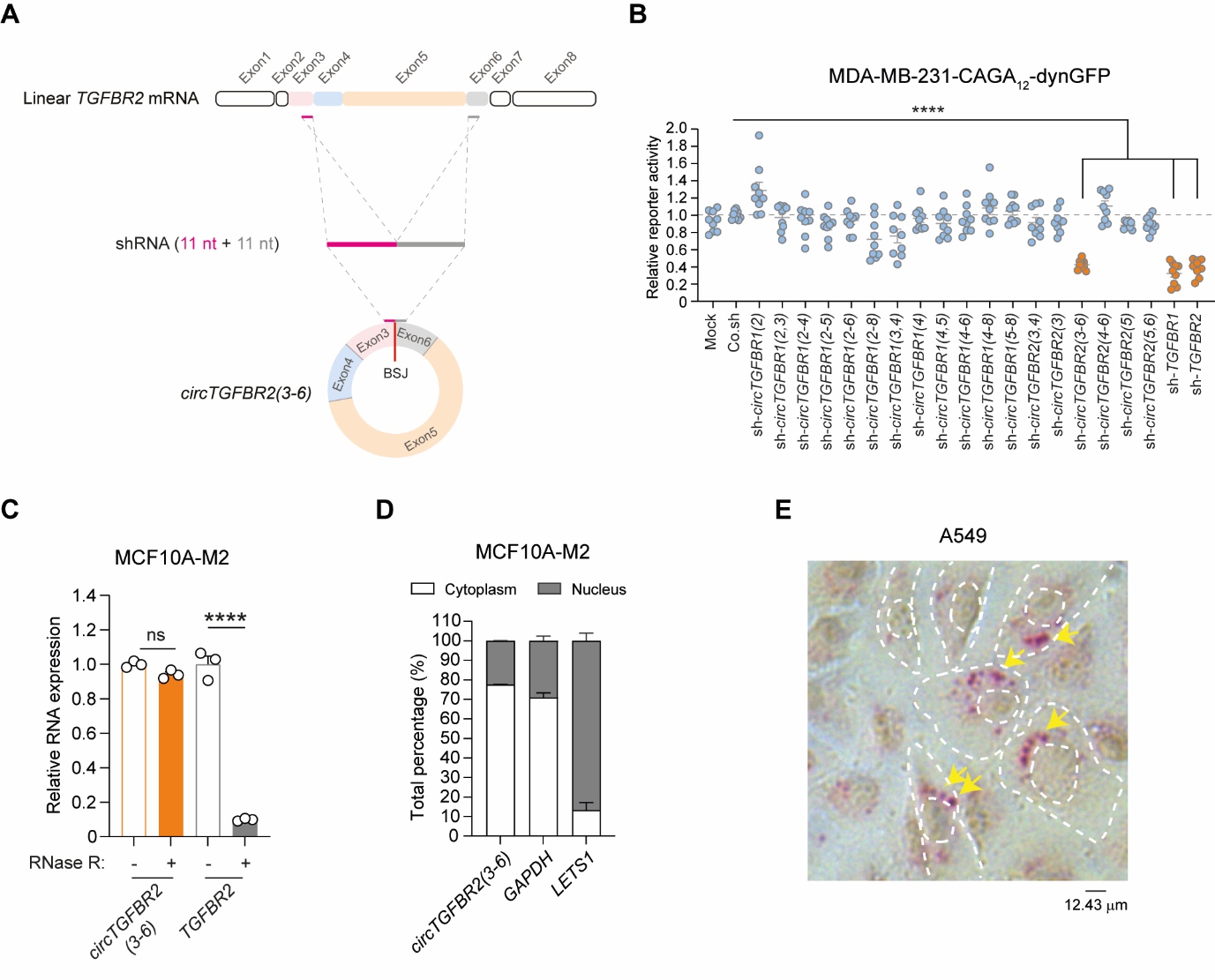


**Supplementary Fig. 1.** **Characterization of *circTGFBR2(3-6)*, an enhancer of TGF-β/SMAD signaling.** (**A**) Schematic representation of shRNA construct targeting the BSJ RNA sequence of circRNA without targeting its linear counterpart. Linear *TGFBR2* mRNA, *circTGFBR2 (3-6)*, and its selective shRNA are shown as an example. (**B**) Representative result of shRNA-mediated circRNA screening in MDA-MB-231 cells stably expressing a SMAD3/4-driven (CAGA)_12_-dynGFP transcriptional reporter. Data are presented as the mean ± SEM from nine biological replicates, with significance assessed using one-way ANOVA followed by Dunnett’s multiple comparisons test. (**C**) RT-qPCR analysis of *circTGFBR2(3-6)* and *TGFBR2* mRNA expression in MCF10A-M2 cells following RNase R treatment. Data present mean ± SEM from three biological replicates. Statistical significance was calculated using one-way ANOVA followed by Tukey’s multiple comparisons test. (**D**) Subcellular localization analysis of *circTGFBR2(3-6)* in MCF10A-M2 cells by RT-qPCR. *LETS1* and *GAPDH* serve as nuclear and cytoplasmic control markers, respectively. Data are presented as mean ± SEM from three biological replicates. (**E**) *In situ* hybridization analysis of *circTGFBR2(3-6)* subcellular localization in A549 cells. Scale bar = 12.43 μm. Cells and nuclei are outlined with white, and yellow arrows indicate *circTGFBR2(3-6)* signals.


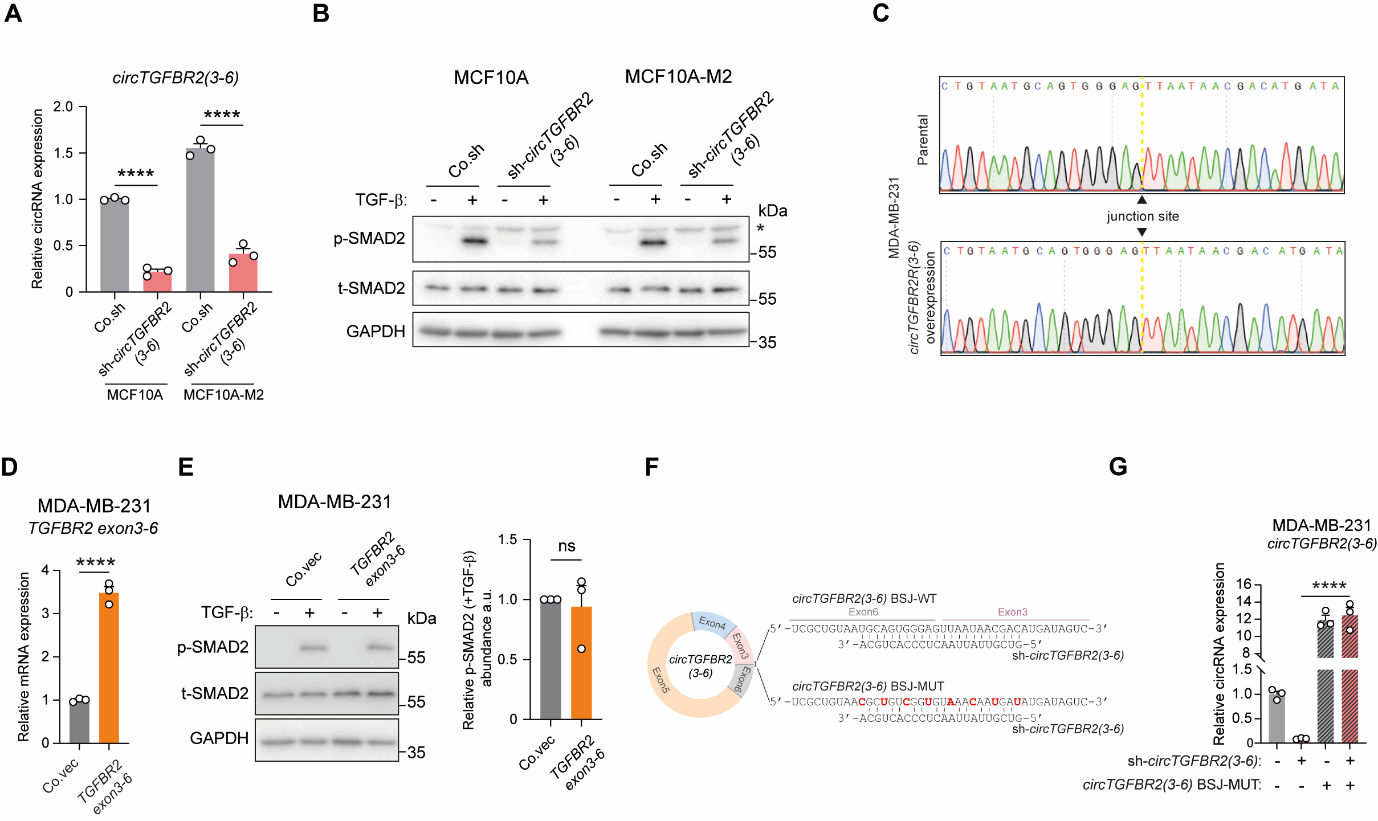


**Supplementary Fig. 2. *circTGFBR2(3-6)*, but not its *TGFBR2 exon3-6* linear counterpart, promotes TGF-β/SMAD signaling.** (**A**) RT-qPCR analysis of shRNA-mediated *circTGFBR2(3-6)* knockdown efficiency in MCF10A and MCF10A-M2 cells. Data present mean ± SEM from three biological replicates. Statistical significance was calculated using one-way ANOVA followed by Tukey’s multiple comparisons test. (**B**) Effect of *circTGFBR2(3-6)* knockdown on TGF-β-induced p-SMAD2 response in MCF10A and MCF10A-M2 cells. GAPDH, loading control. An asterisk (*) indicates a non-specific band. (**C**) Sanger sequencing results confirmed the BSJ sequence of *circTGFBR2(3-6)* in parental MDA-MB-231 cells and those with *circTGFBR2(3-6)* overexpression. (**D**) RT-qPCR analysis of the efficiency of *TGFBR2 exon3-6* ectopic expression in MDA‐MB‐231 cells. Data are presented as the mean ± SEM from three biological replicates, with significance assessed using a two-tailed unpaired Student’s *t*-test. (**E**) Effect of *TGFBR2 exon3-6* ectopic expression on TGF-β-induced p-SMAD2 response in MDA‐MB‐231 cells. Quantitative data represent the relative abundance of p-SMAD2 to t-SMAD2, expressed as mean ± SEM from three independent experiments. GAPDH, loading control. Significance was assessed using a two-tailed paired Student’s *t*-test. (**F**) Schematic representation of the BSJ sequences of endogenous and ectopically expressed shRNA-resistant *circTGFBR2(3-6)*, along with the sequence of *circTGFBR2(3-6)*-specific shRNA. The mutated nucleotides conferring resistance to shRNA are indicated in red. (**G**) RT-qPCR analysis of *circTGFBR2(3-6)* expression in MDA‐MB‐231 cells upon shRNA-mediated endogenous *circTGFBR2(3-6)* knockdown and shRNA-resistant *circTGFBR2(3-6)* overexpression. Data are presented as the mean ± SEM from three biological replicates, with significance assessed using one-way ANOVA followed by Dunnett’s multiple comparisons test.


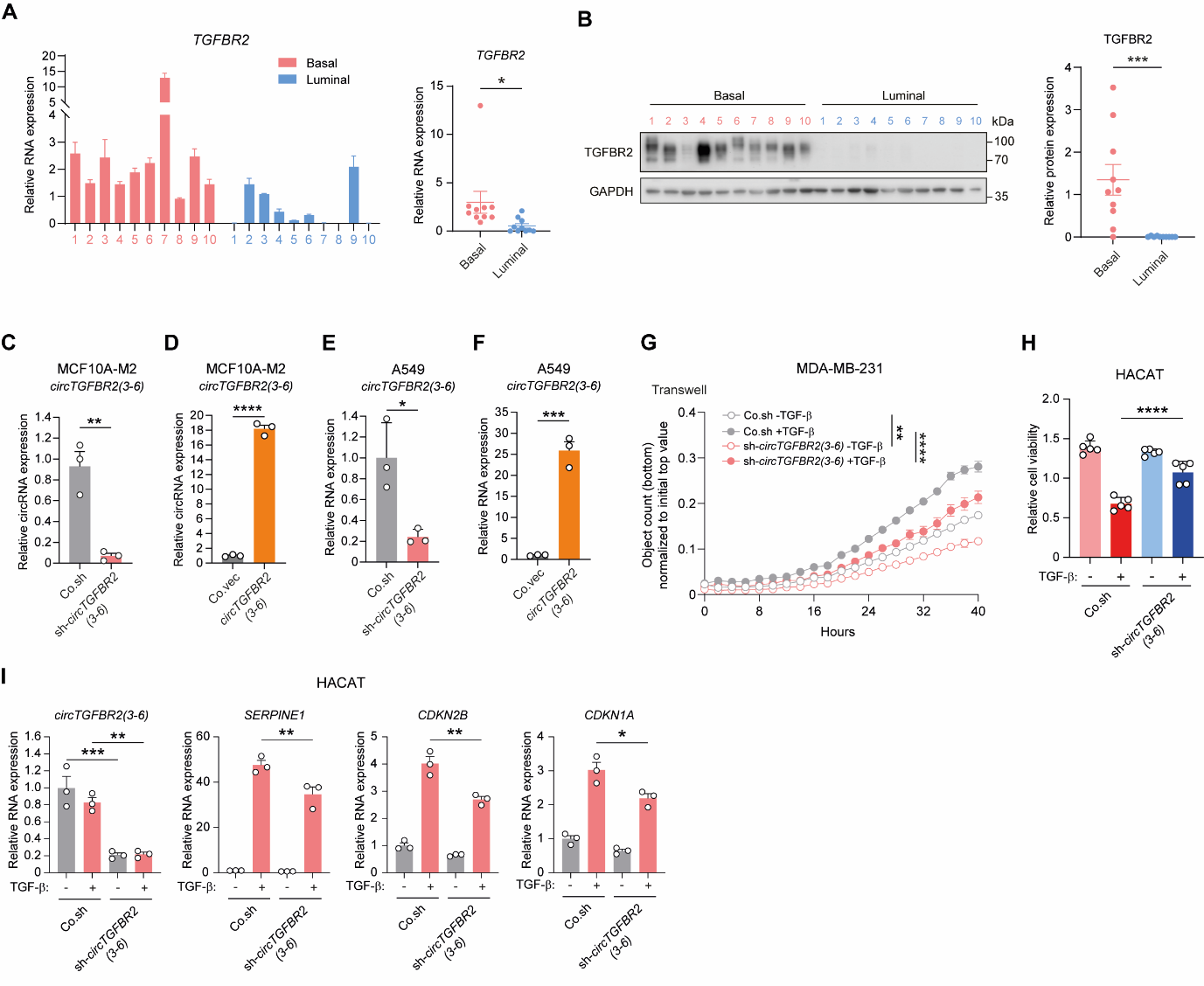


**Supplementary Fig. 3. *circTGFBR2(3-6)* knockdown mitigates TGF-β-induced cellular responses, including TGF-β-induced MDA-MB-231 cell migration and TGF-β-induced inhibition of HACAT cell proliferation and viability.** (**A**) Left: RT-qPCR analysis of *TGFBR2* mRNA expression across the 20 breast cancer cell lines. Data are presented as mean ± SEM from three technical replicates. Right: Comparison of *TGFBR2* mRNA expression between basal-type and luminal-type breast cancer cell lines shown in the left panel. Statistical significance was assessed using a two-tailed unpaired Student’s *t*-test. (**B**) Comparison of TGFBR2 protein expression between basal-type and luminal-type breast cancer cell lines. Statistical significance was assessed using a two-tailed unpaired Student’s *t*-test. GADH, loading control. (**C**, **D**) RT-qPCR analysis of shRNA-mediated *circTGFBR2(3-6)* knockdown efficiency (**C**) and *circTGFBR2(3-6)* ectopic expression efficiency (**D**) in MCF10A-M2 cells. Data are presented as mean ± SEM from three biological replicates. Statistical significance was assessed using a two-tailed unpaired Student’s *t*-test. (**E**, **F**) RT-qPCR analysis of shRNA-mediated *circTGFBR2(3-6)* knockdown efficiency (**E**) and *circTGFBR2(3-6)* ectopic expression efficiency (**F**) in A549 cells. Data are presented as mean ± SEM from three biological replicates. Statistical significance was assessed using a two-tailed unpaired Student’s *t*-test. (**G**) *circTGFBR2(3-6)* knockdown inhibits basal and TGF-β-induced migration in MDA-MB-231 cells as measured using a transwell migration assay. Data are presented as mean ± SEM from nine biological replicates, with significance analyzed using two-way ANOVA followed by Tukey’s multiple comparisons test. (**H**) MTS tetrazolium assay assessing the effect of *circTGFBR2(3-6)* knockdown on TGF-β-induced inhibition of HACAT cell proliferation/viability. Data present mean ± SD from five biological replicates. Statistical significance was assessed using one-way ANOVA followed by Tukey’s multiple comparisons test. (**I**) Effect of *circTGFBR2(3-6)* knockdown on TGF-β-induced expression of *SERPINE1* (encoding PAI-1), *CDKN2B* (encoding p15) and *CDKN1A* (encoding p27) in HACAT cells. RT-qPCR results are presented as mean ± SD from three biological replicates. Statistical significance was assessed using two-way ANOVA followed by Šídák's multiple comparisons test.

**
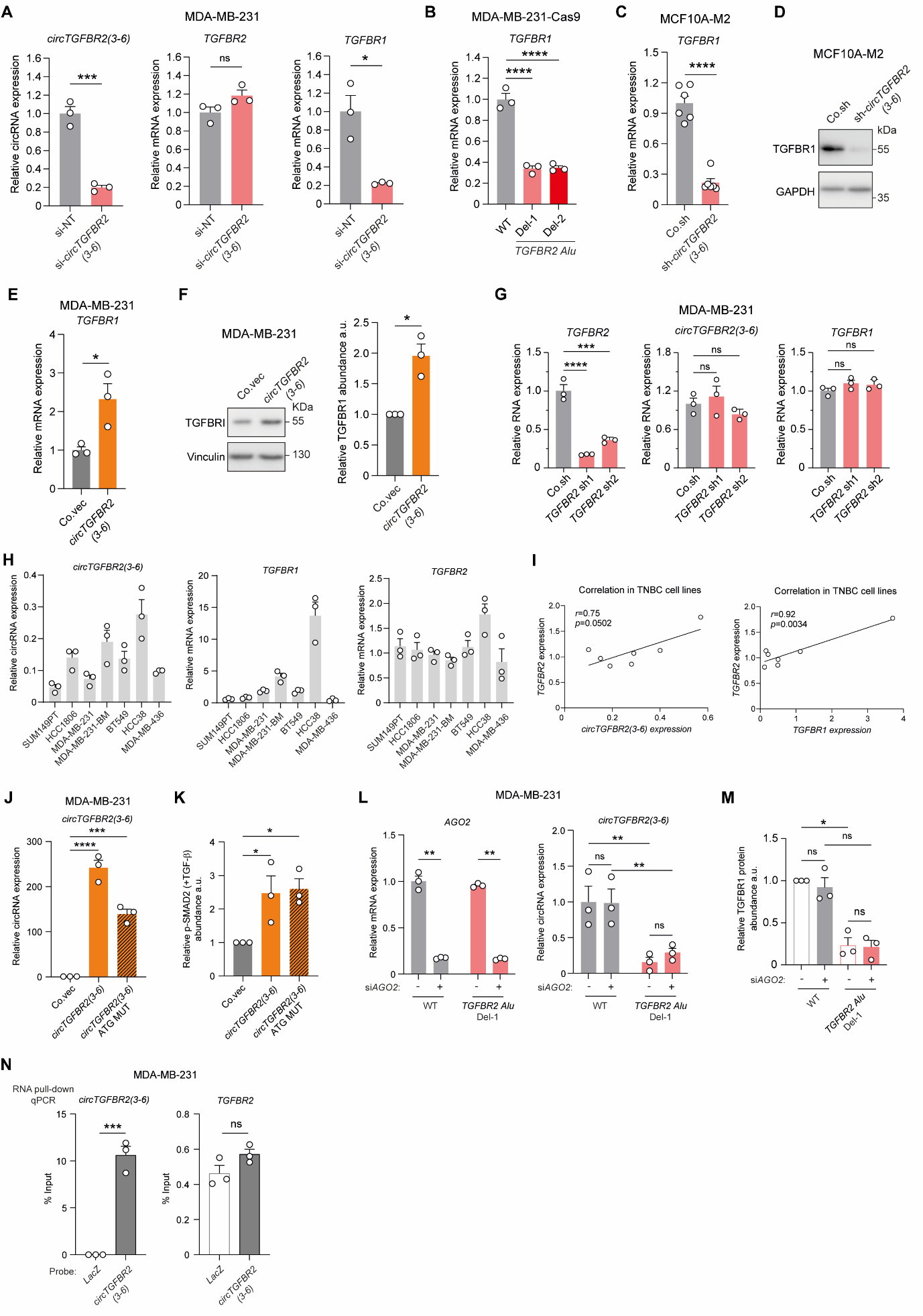
**

**Supplementary Fig. 4. *circTGFBR2(3-6)* binds to and stabilizes *TGFBR1* mRNA.** (**A**) RT-qPCR analysis of *circTGFBR2(3-6)*, *TGFBR2*, and *TGFBR1* mRNA expression in MDA‐MB‐231 cells upon siRNA-mediated *circTGFBR2(3-6)* knockdown. Data are presented as the mean ± SEM from three biological replicates, with significance assessed using a two-tailed unpaired Student’s *t*-test. si-NT, non-targeting siRNA. (**B**) RT-qPCR analysis of genomic *TGFBR2 Alu* deletion-mediated *circTGFBR2(3-6)* knockdown on *TGFBR1* mRNA expression in MDA-MB-231 cells stably expressing Cas9. Data are presented as mean ± SEM from three biological replicates. Statistical significance was determined using one-way ANOVA followed by Dunnett’s multiple comparisons test. (**C**) RT-qPCR analysis of the effect of shRNA-mediated *circTGFBR2(3-6)* knockdown on *TGFBR1* mRNA expression in MCF10A-M2 cells. Data are presented as mean ± SEM from six biological replicates. Statistical significance was assessed using a two-tailed unpaired Student’s *t*-test. (**D**) Effect of shRNA-mediated *circTGFBR2(3-6)* knockdown on TGFBR1 protein expression in MCF10A-M2 cells. GAPDH, loading control. (**E**) RT-qPCR analysis of the effect of *circTGFBR2(3-6)* ectopic expression on *TGFBR1* mRNA expression in MDA-MB-231 cells. Data are presented as mean ± SEM from three biological replicates. Statistical significance was assessed using a two-tailed unpaired Student’s *t*-test. (**F**) Effect of *circTGFBR2(3-6)* ectopic expression on TGFBR1 protein expression in MDA-MB-231 cells. Vinculin, loading control. Data are presented as the mean ± SEM from three independent experiments, with significance assessed using a two-tailed paired Student’s *t*-test. (**G**) RT-qPCR analysis of the effects of *TGFBR2* knockdown using two independent shRNA constructs targeting its 3′UTR on *circTGFBR2(3-6)* and *TGFBR1* mRNA expression in MDA-MB-231 cells. Data are presented as mean ± SEM from three biological replicates. Statistical significance was determined using one-way ANOVA followed by Dunnett’s multiple comparisons test. (**H**) RT-qPCR analysis of *circTGFBR2(3-6)*, *TGFBR1*, and *TGFBR2* mRNA expression across seven triple-negative breast cancer (TNBC) cell lines. Data are presented as the mean ± SEM from three biological replicates. (**I**) Correlations between *TGFBR2* mRNA and either *circTGFBR2(3-6)* or *TGFBR1* mRNA expression across seven TNBC cell lines. Pearson’s *r* and two-tailed *p* value were used to assess correlation. (**J**) RT-qPCR analysis of *circTGFBR2(3-6)* expression in MDA‐MB‐231 cells upon *circTGFBR2(3-6)* and *circTGFBR2(3-6)* ATG MUT ectopic expression. Data are presented as the mean ± SEM from three biological replicates, with significance calculated using one-way ANOVA followed by Dunnett’s multiple comparisons test. (**K**) Quantification of Western blotting analysis of TGF-β-induced p-SMAD2 levels in MDA‐MB‐231 cells upon *circTGFBR2(3-6)* and *circTGFBR2(3-6)* ATG MUT ectopic expression (representative images shown in **Fig. 4G**). Statistical significance was calculated using one-way ANOVA followed by Tukey’s multiple comparisons test. (**L**) RT-qPCR analysis of *AGO2* and *circTGFBR2(3-6)* expression in MDA‐MB‐231 cells (WT and *TGFBR2 Alu* Del-1) upon siRNA-mediated *AGO2* knockdown. Data are expressed as mean ± SEM from three biological replicates, with significance assessed using two-way ANOVA followed by uncorrected Fisher's LSD test. (**M**) Quantification of Western blotting analysis of TGFBR1 protein levels in MDA‐MB‐231 cells (WT and *TGFBR2 Alu* Del-1) upon siRNA-mediated *AGO2* knockdown (representative images shown in **Fig. 4I**). Statistical significance was calculated using one-way ANOVA followed by Tukey’s multiple comparisons test. (**N**) RNA pull-down assay using a probe targeting *circTGFBR2(3-6)* in MDA‐MB‐231 cells. A *LacZ*-targeting probe served as a control. RT-qPCR detected *circTGFBR2(3-6)* and *TGFBR2* mRNA in immunoprecipitates. Data represent mean ± SEM from three biological replicates, with significance assessed using a two-tailed unpaired Student’s *t*-test.

**
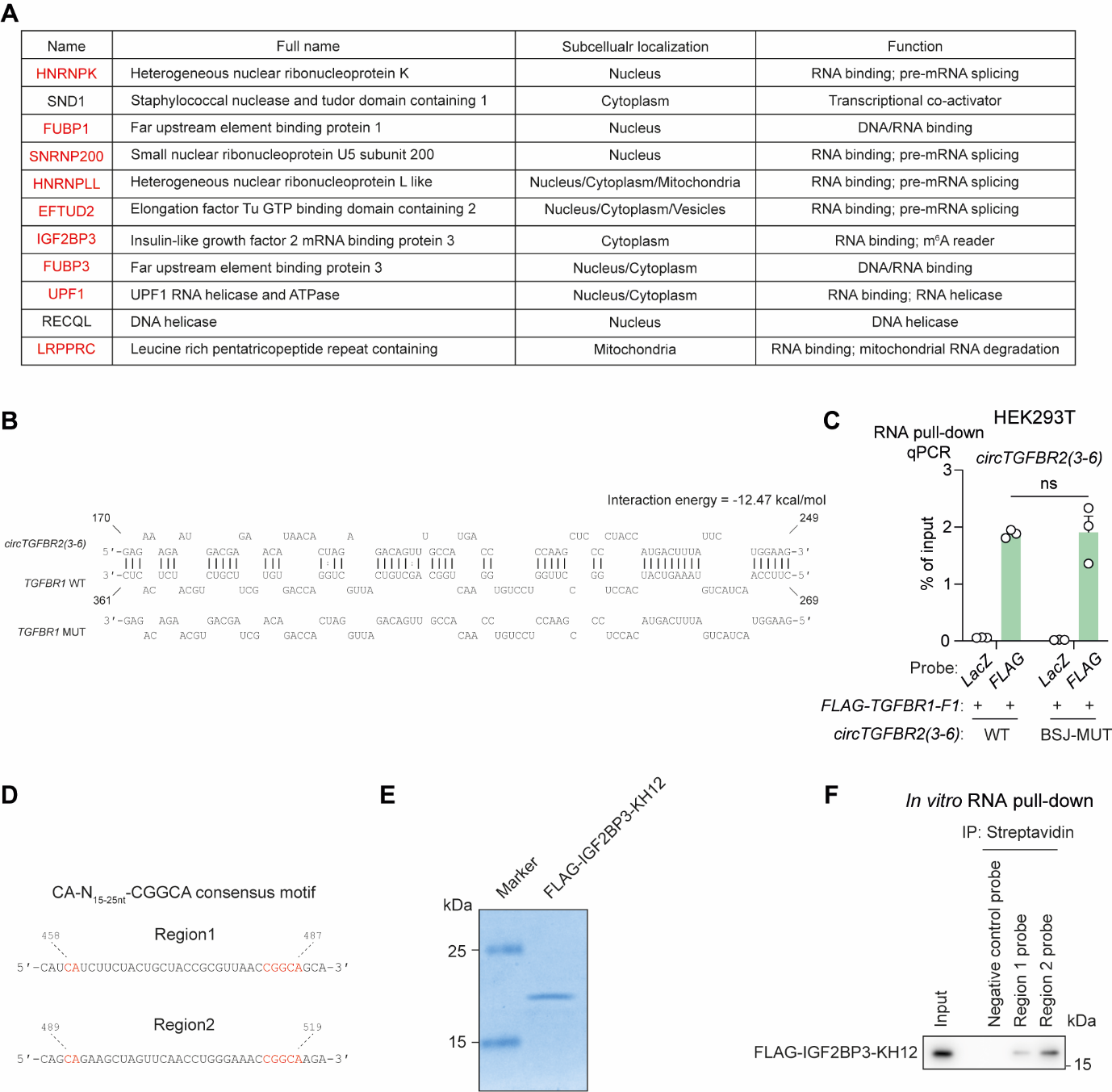
**

**Supplementary Fig. 5. *circTGFBR2(3-6)* scaffolds IGF2BP3 and *TGFBR1* mRNA.** (**A**) List of *circTGFBR2(3-6)*-interacting proteins and their functions. Subcellular localization information is obtained from the Human Protein Atlas (<https://www.proteinatlas.org/>). RNA-binding proteins are marked in red. (**B**) Schematic of the predicted interaction between *circTGFBR2(3-6)* and *TGFBR1* mRNA. The complementary binding region is shown, along with the sequence of the mutated *TGFBR1* mRNA (*TGFBR1* MUT) in which the predicted interaction site has been disrupted. (**C**) Interactions between *FLAG-TGFBR1-F1* and either WT or BSJ-MUT *circTGFBR2(3-6)* in HEK293T cells, analyzed by RNA pull-down followed by RT-qPCR. Data represent mean ± SEM from three biological replicates, with significance assessed using a two-tailed unpaired Student’s *t*-test. (**D**) Schematic representation of the two regions within *circTGFBR2(3-6)* comprising the CA-N_15-25nt_-CGGCA RNA consensus motif. (**E**) Representative image of SDS-polyacrylamide gel stained with Coomassie, showing the purified FLAG-IGF2BP3-KH12 protein. Protein markers are shown on the left side of the gel. (**F**) *In vitro* RNA pull-down analysis of the interactions between the purified FLAG-IGF2BP3-KH12 protein and either region 1 or region 2 probes of *circTGFBR2(3-6)*. Western blotting detected FLAG expression in input (on left side) and immunoprecipitates.

**
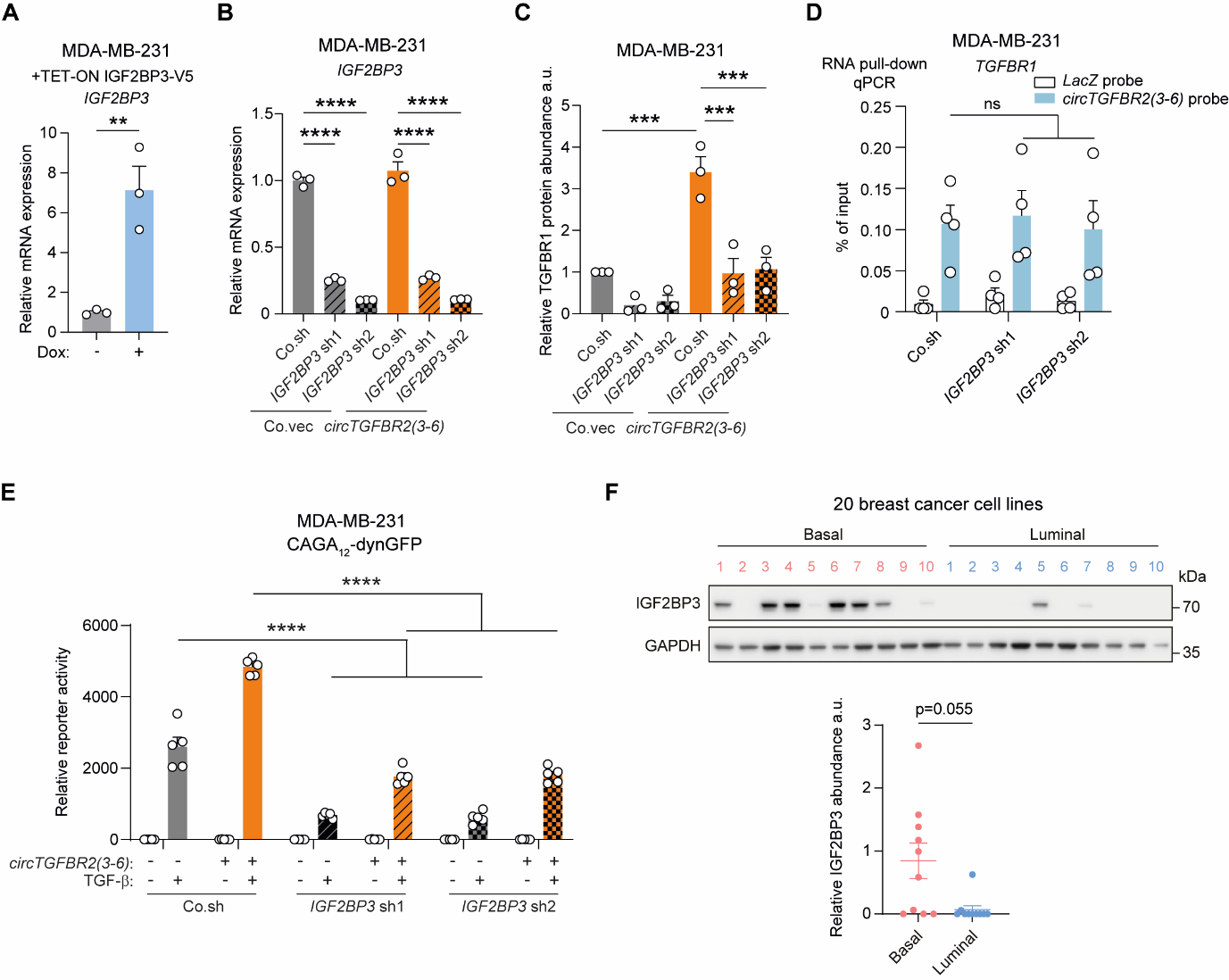
** **Supplementary Fig. 6. IGF2BP3 promotes *TGFBR1* expression and TGF**-**β/SMAD signaling.** (**A**) RT-qPCR analysis of *IGF2BP3* mRNA expression in MDA‐MB‐231 cells upon IGF2BP3 ectopic expression using a TET-ON inducible system. Data are presented as mean ± SEM from three biological replicates, with significance analyzed using a two-tailed unpaired Student’s *t*-test. (**B**) RT-qPCR analysis of *IGF2BP3* mRNA expression in MDA‐MB‐231 cells upon *IGF2BP3* knockdown and *circTGFBR2(3-6)* ectopic expression. Data are presented as mean ± SEM from three biological replicates, with significance assessed using one-way ANOVA followed by Tukey’s multiple comparisons test. (**C**) Quantification of Western blotting analysis of TGFBR1 relative protein expression in MDA‐MB‐231 cells upon *IGF2BP3* knockdown and *circTGFBR2(3-6)* ectopic expression (representative images shown in **Fig. 6H**). Statistical significance was calculated using one-way ANOVA followed by Tukey’s multiple comparisons test. (**D**) Interaction between *circTGFBR2(3-6)* and *TGFBR1* mRNA in MDA-MB-231 cells upon *IGF2BP3* knockdown, as analyzed by RNA pull-down followed by RT-qPCR. Data represent mean ± SEM from four independent experiments, with significance assessed using two-way ANOVA followed by Šídák's multiple comparisons test. (**E**) Effect of *circTGFBR2(3-6)* ectopic expression and *IGF2BP3* knockdown on the TGF-β-induced CAGA_12_-dynGFP reporter activity in MDA‐MB‐231 cells. Data are presented as mean ± SEM from five biological replicates. Statistical significance was assessed using two-way ANOVA followed by Šídák's multiple comparisons test. (**F**) Comparison of IGF2BP3 protein expression between the basal-type and luminal-type breast cancer cell lines. GAPDH, loading control. Statistical significance was assessed using a two-tailed unpaired Student’s *t*-test.

**
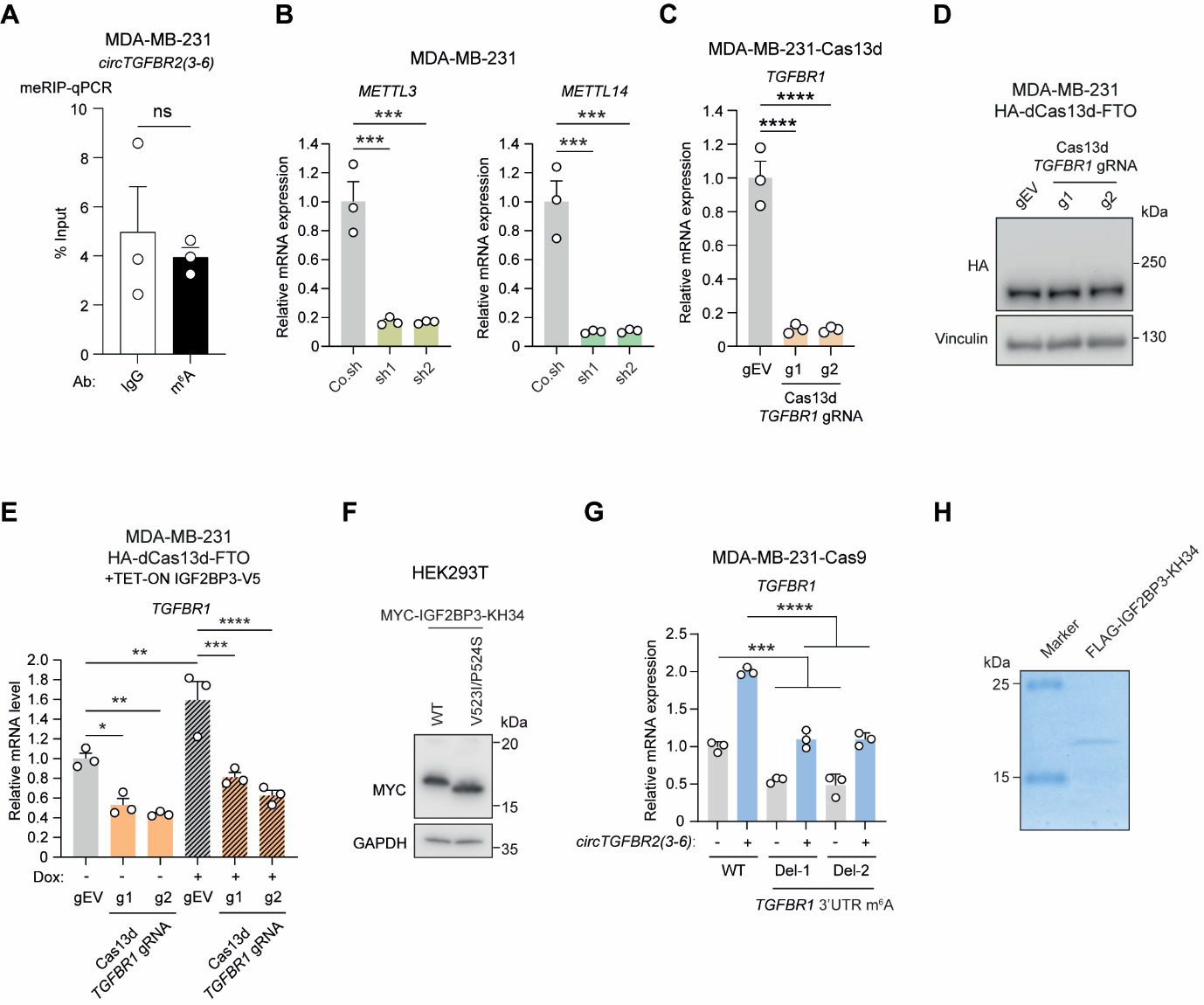
**

**Supplementary Fig. 7. IGF2BP3 binds to and stabilizes m^6^A-modified *TGFBR1* mRNA.** (**A**) m^6^A abundance on *circTGFBR2(3-6)* in MDA-MB-231 cells, as analyzed by meRIP. RT-qPCR was performed to detect *circTGFBR2(3-6)* in immunoprecipitates. Data are presented as mean ± SEM from three biological replicates, with significance assessed using a two-tailed unpaired Student’s *t*-test. (**B**) Efficiency of shRNA-mediated *METTL3* and *METTL14* knockdown in MDA‐MB‐231 cells, analyzed by RT-qPCR. Data are presented as mean ± SEM from three biological replicates, with significance analyzed using one-way ANOVA followed by Dunnett’s multiple comparisons test. (**C**) The efficiency of *TGFBR1* knockdown using the Cas13d system in MDA‐MB‐231 cells was analyzed by RT-qPCR. Data are presented as mean ± SEM from three biological replicates, with significance analyzed using one-way ANOVA followed by Dunnett’s multiple comparisons test. (**D**) Western blotting validation of HA-dCas13d-FTO expression in MDA-MB-231 stable cells. (**E**) RT-qPCR analysis of *TGFBR1* mRNA in MDA‐MB‐231 cells upon IGF2BP3 ectopic expression (using a TET-ON inducible system) and m^6^A removal from *TGFBR1* mRNA using the dCas13d-FTO eraser system. Data are presented as mean ± SEM from three biological replicates, with significance analyzed using one-way ANOVA followed by Tukey’s multiple comparisons test. (**F**) Western blotting validation of WT or MUT (V523I/P524S) MYC-IGF2BP3-KH34 expression in HEK293T cells. GAPDH, loading control. (**G**) Effect of *circTGFBR2(3-6)* ectopic expression and *TGFBR1* 3′UTR m^6^A deletion on *TGFBR1* mRNA expression in MDA‐MB‐231 cells. RT-qPCR data are presented as mean ± SEM from three biological replicates, with significance analyzed using one-way ANOVA followed by Dunnett’s multiple comparisons test. (**H**) Representative image of SDS-polyacrylamide gel stained with Coomassie, showing the purified FLAG-IGF2BP3-KH34 protein. Protein markers are shown on the left side of the gel.

**Supplementary Table 1. Sequences of the predicted *Alu* DNA elements.**

| Intron2 *Alu* | GGCCAAACATGGACTCCTTGTATTCATCCTGTGTTCACTCATTTATCCAATAAACATTTTTGTATGCCTGCTATGTGAAAGGTTCTCTTCCTGGCACTGGAGATATAGTAGGTAAGTGAGGTGGAGAAAGTTTCATGGAGCTTACATTTTTGTGGATAGATTATTAGCAAGTATGCGATGCAGATAGTTTGTGCAGACAATAAATAAAACAGAGTTGCTATAGGATGAAGAGGTGGCATTTGATCAGAGATCAGAAAAGTGACAGGAAGCTAAACAAAGGTAATAGCAAGTGAAAGGCCTGAGGAGGGAATGAACTTGGCTTCTTCCCAGAAGGGGGCAGGAGATTGGTGATGGTGGTGATAAGGGAGAGGTACAGAAGGCAGATCCTGAGGTGGATTCAGCTTTTATTTGCTGCGATCATAACTCCAATGCCTCACCAAT |
| --- | --- |
| Intron7 *Alu* | GACAGGGTCTTAGGTCTCACCTGAGAAATCTGTGCACCCAAGTTCATCCTGGTTGGCTGCCAAATGAAGCCAAACTAACTCCACCCTTTTTTTTTTTTTGAGACAGTCTCGCTCTGTCACCCAGGCTGGAGTGCAATGGCGCAATCTCGGCTGACTGCAACCTCTGCCTCCCGGGTTCAAGCAATTCTCCTAGCTCAGCCTCTTGAGTAGCTGAGATTACAGACATGCACCACCACGCTCAGCTAATATTTGTATTTTTAGTAGAGACAGGGTTACACCGTGTTGATCAGGCTGATATCGAACTCTTGACCTCATGATCTGCCCACCTCGGCCTCCCAAAGTGCTGGGATTACAGGCGTGAGCCACTGCACCCAGCCTCCATTCCATGTTTAACTTTTTGTTTTGTGATAATTTTAGACTTAGAAAA |

**Supplementary Table 2. Primers that were used for molecular cloning.**

| Gene | Sequence (5' to 3') | |
| --- | --- | --- |
| *circTGFBR2(3-6)* | Forward | GCGTCTCATCAGTTAATAACGACATGATAGTCACTGACAAC |
|  | Reverse | TCGTCTCATTACCTCCCACTGCATTACAGCG |
| *TGFBR2(3-6) linear* | Forward | GATTCTAGATTAATAACGACATGATAGTCACTGACAAC |
|  | Reverse | TAGCGAATTCTTACCTCCCACTGCATTACAG |
| *circTGFBR2(3-6) shRNA resistant* | Forward | GCGTCTCATCAGTAAACAATGATATGATAGTCACTGACAACAACGGTG |
|  | Reverse | TCGTCTCATTACCACCGACAGCGTTACAGCGAGATGTCATTTCCCAG |
| sh-*circTGFBR2(3-6)* | Sense | CCGGTGCAGTGGGAGTTAATAACGACTCGAGTCGTTATTAACTCCCACTGCATTTTTG |
|  | Antisense | AATTCAAAAATGCAGTGGGAGTTAATAACGACTCGAGTCGTTATTAACTCCCACTGCA |
| *circTGFBR2(3-6)* KO g1 | Sense | ACCGAAGCATAGAGCTCTTGGTCT |
|  | Antisense | AAACAGACCAAGAGCTCTATGCTT |
| *circTGFBR2(3-6)* KO g2 | Sense | ACCGAAGCTACAAAGACAGTTGCA |
|  | Antisense | AAACTGCAACTGTCTTTGTAGCTT |
| *circTGFBR2(3-6)* KO g3 | Sense | ACCGTAATAAAATCCATTGTACCG |
|  | Antisense | AAACCGGTACAATGGATTTTATTA |
| Cas13d *TGFBR1* g1 | Sense | AAACTAGTGCACAGAAAGGACCCACAT |
|  | Antisense | CTTGATGTGGGTCCTTTCTGTGCACTA |
| Cas13d *TGFBR1* g2 | Sense | AAACCCAAATTAAAACCCAGGAGCAG |
|  | Antisense | CTTGCTGCTCCTGGGTTTTAATTTGG |
| IGF2BP3 FL | Forward | GGGCGAATTCAACAAACTGTATATCGGAAACCTCAGC |
|  | Reverse | GCCGCTCGAGTCACTTCCGTCTTGACTGAGGTGGT |
| IGF2BP3 RRM12 | Forward | GGGCGAATTCAACAAACTGTATATCGGAAACCTCAGC |
|  | Reverse | GCCGCTCGAGTCAGGCGGCCATTTCATCAGGGATATA |
| IGF2BP3 KH12 | Forward | GGGCGAATTCCAGAAACCATGTGATTTGCCTCTGC |
|  | Reverse | GCCGCTCGAGTCATTTCTTCATGATCTCCTCCTCAGCT |
| IGF2BP3 KH34 | Forward | GGGCGAATTCCAATCAGAAACGGAGACTGTTCATCTG |
|  | Reverse | GCCGCTCGAGTCATTCCTGAATTTTTCTCTGGGCAACC |
| *TGFBR1 F1* | Forward | GGGCGAATTCATGGAGGCGGCGGTCGCTGCTC |
|  | Reverse | GCCGCTCGAGGGGTCCTCTTCATTTGGCACTCGATGGT |
| *TGFBR1 F2* | Forward | GGGCGAATTCGAGTGCCAAATGAAGAGGACCC |
|  | Reverse | GCCGCTCGAGCTCTATGAGCAATGGCTGGCTTTC |
| *TGFBR1 F3* | Forward | TGCAGATATCGAAAGCCAGCCATTGCTCATAGAG |
|  | Reverse | TCGAGCGGCCGCGAGAGTTCAGGCAAAGCTGTAGAA |
| *TGFBR1 F4* | Forward | ACTGGCGGCCGCTTCTACAGCTTTGCCTGAACTCT |
|  | Reverse | CATGCTCGAGCAACAAAAGCTTCATATCCTGGTG |
| *circTGFBR2(3-6)* divergent PCR | Forward | CTGGTTGTCACAGGTGGAAAATCTC |
|  | Reverse | GGCTCCAGAAGTCCTAGAATCCA |
| *circTGFBR2(3-6)* covergent PCR | Forward | AAGCAGAACACTTCAGAGCAGTT |
|  | Reverse | CTCATGCTTCAGATTGATGTCTGAGA |
| *circTGFBR2(3-6)* PCR from ECP | Forward | GCGTCTCATCAGTTAATAACGACATGATAGTCACTGACAAC |
|  | Reverse | TCGTCTCATTACCTCCCACTGCATTACAGCG |
| *TGFBR2 intron 2* PCR | Forward | ACCCAAGCTTCACACAGTTCAGAGAGTATTTCTGCC |
|  | Reverse | GCTCGGATCCCATGTCGTTATTAACTGAGGAGAGAG |
| sh-*circTGFBR1(2)* | Sense | CCGGTCCAACTACTGCGTTACAGTGTTTCGAAACACTGTAACGCAGTAGTTGGATTTTTG |
|  | Antisense | AATTCAAAAATCCAACTACTGCGTTACAGTGTTTCGAAACACTGTAACGCAGTAGTTGGA |
| sh-*circTGFBR1(2,3)* | Sense | CCGGTTCTGGCTCAGCGTTACAGTGTTTCGAAACACTGTAACGCTGAGCCAGAATTTTTG |
|  | Antisense | AATTCAAAAATTCTGGCTCAGCGTTACAGTGTTTCGAAACACTGTAACGCTGAGCCAGAA |
| sh-*circTGFBR1(2-4)* | Sense | CCGGAGACAATAAAGCGTTACAGTGTTTCGAAACACTGTAACGCTTTATTGTCTTTTTTG |
|  | Antisense | AATTCAAAAAAGACAATAAAGCGTTACAGTGTTTCGAAACACTGTAACGCTTTATTGTCT |
| sh-*circTGFBR1(2-5)* | Sense | CCGGTGGTACCCAAGCGTTACAGTGTTTCGAAACACTGTAACGCTTGGGTACCATTTTTG |
|  | Antisense | AATTCAAAAATGGTACCCAAGCGTTACAGTGTTTCGAAACACTGTAACGCTTGGGTACCA |
| sh-*circTGFBR1(2-6)* | Sense | CCGGGGAACAAAAAGGGGAACAAAAAGTCGACTTTTTGTTCCCCTTTTTGTTCCTTTTTG |
|  | Antisense | AATTCAAAAAGGAACAAAAAGGGGAACAAAAAGTCGACTTTTTGTTCCCCTTTTTGTTCC |
| sh-*circTGFBR1(2-8)* | Sense | CCGGAGAGCTGTGAACGTTACAGTGTTCGAACACTGTAACGTTCACAGCTCTTTTTTG |
|  | Antisense | AATTCAAAAAAGAGCTGTGAACGTTACAGTGTTCGAACACTGTAACGTTCACAGCTCT |
| sh-*circTGFBR1(3,4)* | Sense | CCGGAGACAATAAAGTAAAGTCATCACTCGAGTGATGACTTTACTTTATTGTCTTTTTTG |
|  | Antisense | AATTCAAAAAAGACAATAAAGTAAAGTCATCACTCGAGTGATGACTTTACTTTATTGTCT |
| sh-*circTGFBR1(4)* | Sense | CCGGAGACAATAAAGGTTTACCATTGCTCGAGCAATGGTAAACCTTTATTGTCTTTTTTG |
|  | Antisense | AATTCAAAAAAGACAATAAAGGTTTACCATTGCTCGAGCAATGGTAAACCTTTATTGTCT |
| sh-*circTGFBR1(4,5)* | Sense | CCGGTGGTACCCAAGGTTTACCATTGCTCGAGCAATGGTAAACCTTGGGTACCATTTTTG |
|  | Antisense | AATTCAAAAATGGTACCCAAGGTTTACCATTGCTCGAGCAATGGTAAACCTTGGGTACCA |
| sh-*circTGFBR1(4-6)* | Sense | CCGGGGAACAAAAAGGTTTACCATTGCTCGAGCAATGGTAAACCTTTTTGTTCCTTTTTG |
|  | Antisense | AATTCAAAAAGGAACAAAAAGGTTTACCATTGCTCGAGCAATGGTAAACCTTTTTGTTCC |
| sh-*circTGFBR1(4-8)* | Sense | CCGGAGAGCTGTGAAGTTTACCATTGCTCGAGCAATGGTAAACTTCACAGCTCTTTTTTG |
|  | Antisense | AATTCAAAAAAGAGCTGTGAAGTTTACCATTGCTCGAGCAATGGTAAACTTCACAGCTCT |
| sh-*circTGFBR1(5-8)* | Sense | CCGGAGAGCTGTGAAACAATGGTACTTTCGAAAGTACCATTGTTTCACAGCTCTTTTTTG |
|  | Antisense | AATTCAAAAAAGAGCTGTGAAACAATGGTACTTTCGAAAGTACCATTGTTTCACAGCTCT |
| sh-*circTGFBR2(3,4)* | Sense | CCGGCATCTTCTCAGAAGTTAATAACTCGAGTTATTAACTTCTGAGAAGATGTTTTTG |
|  | Antisense | AATTCAAAAACATCTTCTCAGAAGTTAATAACTCGAGTTATTAACTTCTGAGAAGATG |
| sh-*circTGFBR2(3)* | Sense | CCGGGTGGCTGTATGTTAATAACGACATCGATGTCGTTATTAACATACAGCCACTTTTTG |
|  | Antisense | AATTCAAAAAGTGGCTGTATGTTAATAACGACATCGATGTCGTTATTAACATACAGCCAC |
| sh-*circTGFBR2(4-6)* | Sense | CCGGGTGGGAGGAGAAAGAATGACGTCGACGTCATTCTTTCTCCTCCCACTTTTTG |
|  | Antisense | AATTCAAAAAGTGGGAGGAGAAAGAATGACGTCGACGTCATTCTTTCTCCTCCCAC |
| sh-*circTGFBR2(5)* | Sense | CCGGACAGTGGGCAGAATATAACACCATCGATGGTGTTATATTCTGCCCACTGTTTTTTG |
|  | Antisense | AATTCAAAAAACAGTGGGCAGAATATAACACCATCGATGGTGTTATATTCTGCCCACTGT |
| sh-*circTGFBR2(5,6)* | Sense | CCGGTGCAGTGGGAGAATATAACACCTCGAGGTGTTATATTCTCCCACTGCATTTTTG |
|  | Antisense | AATTCAAAAATGCAGTGGGAGAATATAACACCTCGAGGTGTTATATTCTCCCACTGCA |
| *TGFBR1 3’UTR* KO g1 | Sense | ACCGTTGGAGCCAGAACACTGCCA |
|  | Antisense | AAACTGGCAGTGTTCTGGCTCCAA |
| *TGFBR1 3’UTR* KO g2 | Sense | ACCGTGGATTACTGGAATACCCAT |
|  | Antisense | ACATGGGTATTCCAGTAATCCA |
| *TGFBR1 3’UTR* KO PCR | Forward | GATGGCAAAGAGATCGTTAGAGTG |
|  | Reverse | TATCAAACACCCTATTCCGTTGG |

**Supplementary Table 3. Primers that were used for RT-qPCR.**

| Gene | Sequence (5' to 3') | |
| --- | --- | --- |
| *circTGFBR2(3-6)* | Forward | CTGGTTGTCACAGGTGGAAAATCTC |
|  | Reverse | GGCTCCAGAAGTCCTAGAATCCA |
| *TGFBR2* | Forward | GCACGTTCAGAAGTCGGATG |
|  | Reverse | GGATGGGCAGTCCTATTACAGC |
| *LETS1* | Forward | AGATTGCACAGCACTGGTGA |
|  | Reverse | GAGGCAAGCTAACACCCACA |
| *SERPINE1* | Forward | CACAAATCAGACGGCAGCACT |
|  | Reverse | CATCGGGCGTGGTGAACTC |
| *CCN2* | Forward | TTGCGAAGCTGACCTGGAAGAGAA |
|  | Reverse | AGCTCGGTATGTCTTCATGCTGGT |
| *SNAI1* | Forward | ACCACTATGCCGCGCTCTT |
|  | Reverse | GGTCGTAGGGCTGCTGGAA |
| *TGFBR1* | Forward | ACGGCGTTACAGTGTTTCTG |
|  | Reverse | GCACATACAAACGGCCTATCT |
| *AGO2* | Forward | ACCATGTACTCGGGAGCCG |
|  | Reverse | GGTGGAGGCTTGAAGGCATA |
| *FLAG* | Forward | GAACCCACTGCTTACTGGCTTATC |
|  | Reverse | GAATTCGCCCTTGTCATCATCGT |
| *IGF2BP3* | Forward | CGGTCCCAAAAAGGCAAAGG |
|  | Reverse | ACTATCCAGCACCTCCCACT |
| *METTL3* | Forward | CAAGGCTTCAACCAGGGTCT |
|  | Reverse | GGTTTCCAAGGGTGATCCAGT |
| *METTL14* | Reverse | TGTCTTTCAGAGAACAAAGGAACAC |
|  | Reverse | CGTCTTCTACCAAGACAAAAATGCT |
| *GAPDH* | Forward | TGCACCACCAACTGCTTAGC |
|  | Reverse | GGCATGGACTGTGGTCATGAG |
| *CDKN2B* | Forward | TCCAGGGGTCGTTTGCTTTT |
|  | Reverse | GCAGACATTGGAGTGAACGC |
| *CDKN1A* | Forward | CCTCATCCCGTGTTCTCCTTT |
|  | Reverse | GTACCACCCAGCGGACAAGT |

**Supplementary Table 4. Antibodies that were used for Western blotting.**

| Antibody | Company | Catalog number | Dilution |
| --- | --- | --- | --- |
| Phospho-SMAD2 | Cell Signaling Technology | 3108 | 1:1000 |
| Total SMAD2 | Epitomics | 1736-1 | 1:1000 |
| TGFBR1 | Abcam | ab235578 | 1:1000 |
| TGFBR2 | Abcam | ab259360 | 1:1000 |
| E-cadherin | BD | 610181 | 1:1000 |
| N-cadherin | BD | 610920 | 1:1000 |
| Vimentin | Cell Signaling Technology | 5741 | 1:5000 |
| Fibronectin | Sigma-Aldrich | F7387 | 1:500 |
| IGF2BP3 | Abcam | ab177477 | 1:1000 |
| FLAG | Sigma-Aldrich | F7425 | 1:1000 |
| V5 | Sigma-Aldrich | V8012 | 1:1000 |
| MYC | Sigma-Aldrich | M4439 | 1:1000 |
| HA | Santa Cruz | sc805 | 1:1000 |
| Vinculin | Sigma-Aldrich | V9131 | 1:5000 |
| GAPDH | Merck Millipore | MAB374 | 1:5000 |

**Supplementary Table 5. Probes that were used for *in vitro* RNA pull-down.**

| Probe | Sequence (5' to 3') |
| --- | --- |
| Negative control | GUGUAUUUUGGAGGAACUACAGAAUGGCCGU |
| *circTGFBR2(3-6)* region 1 | CAUCAUCUUCUACUGCUACCGCGUUAACCGGCAGCA |
| *circTGFBR2(3-6)* region 2 | CAGCAGAAGCUGAGUUCAACCUGGGAAACCGGCAAGA |
| *TGFBR1* 3′UTR | CUUUGGACAUG |
| *TGFBR1* 3′UTR-m^6^A | CUUUGG(m^6^A)CAUG |
