# Supplementary figures and images for: circTGFBR2(3-6) acts as an assembly platform for RNA-binding protein IGF2BP3 and TGFBR1 mRNA to enhance breast cancer cell plasticity
Source: Cell Death Differ. 2025 Oct 27;33(4):779–97. doi: 10.1038/s41418-025-01597-2 (PMC13077047; doi:10.1038/s41418-025-01597-2)

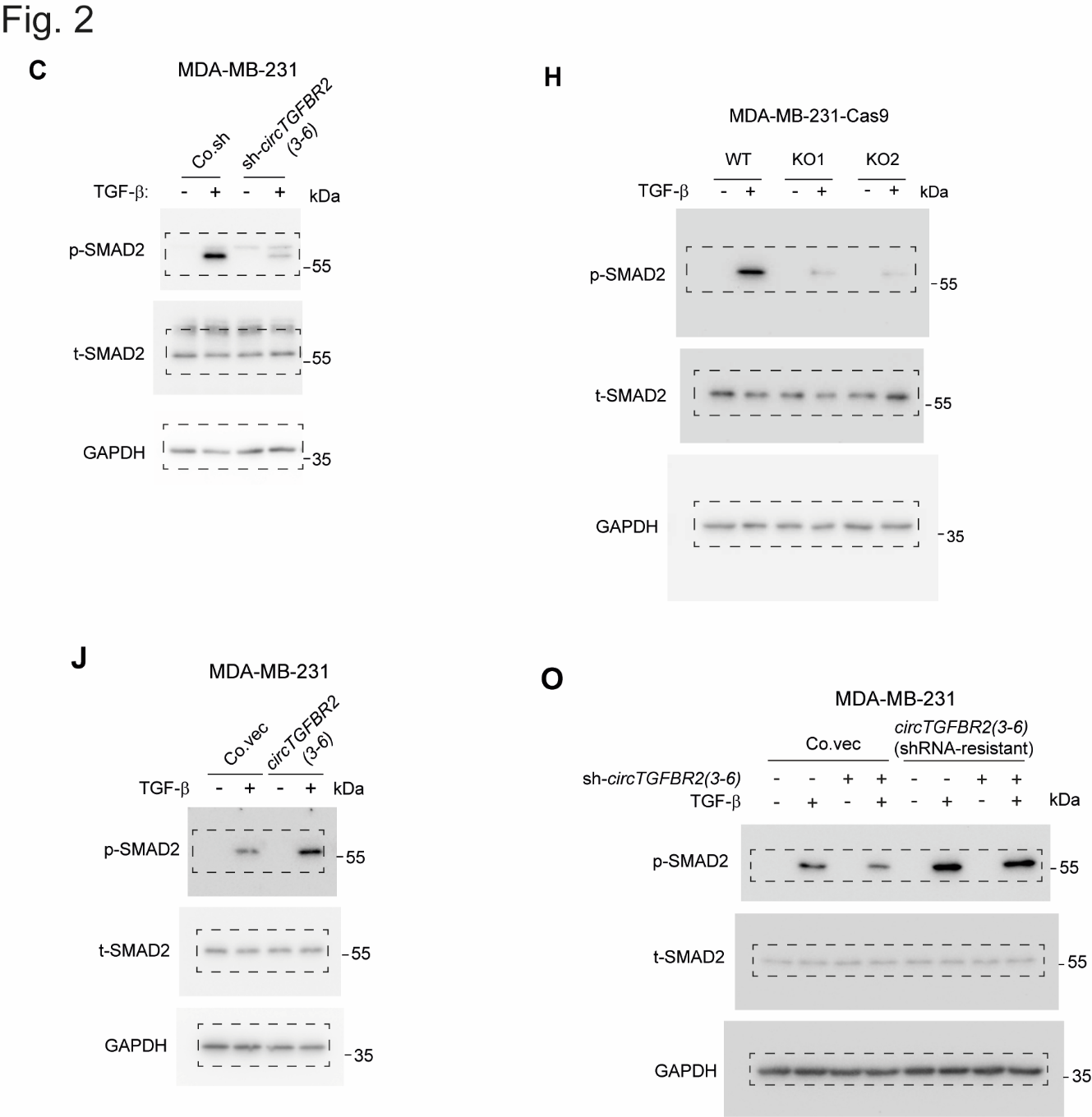

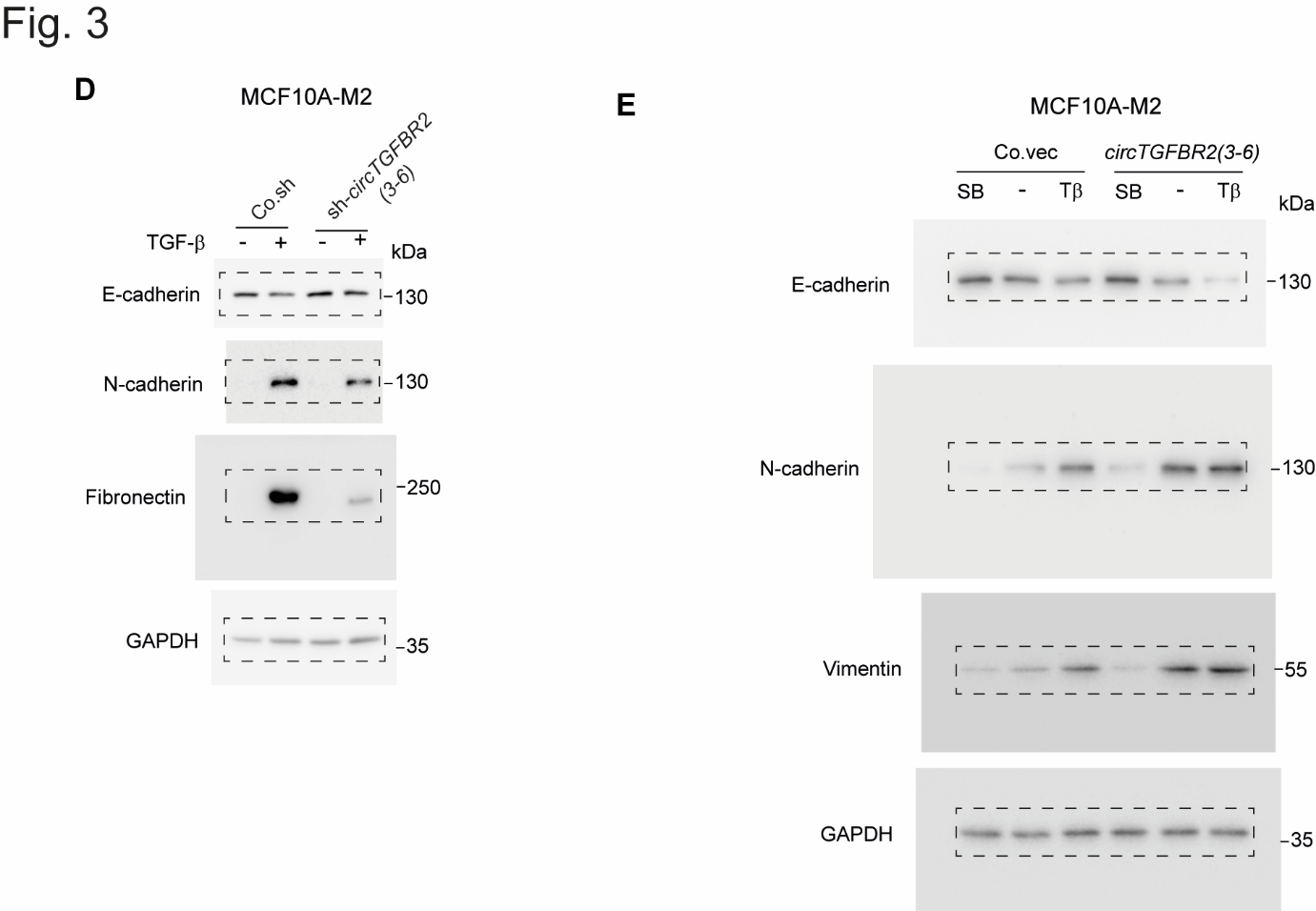

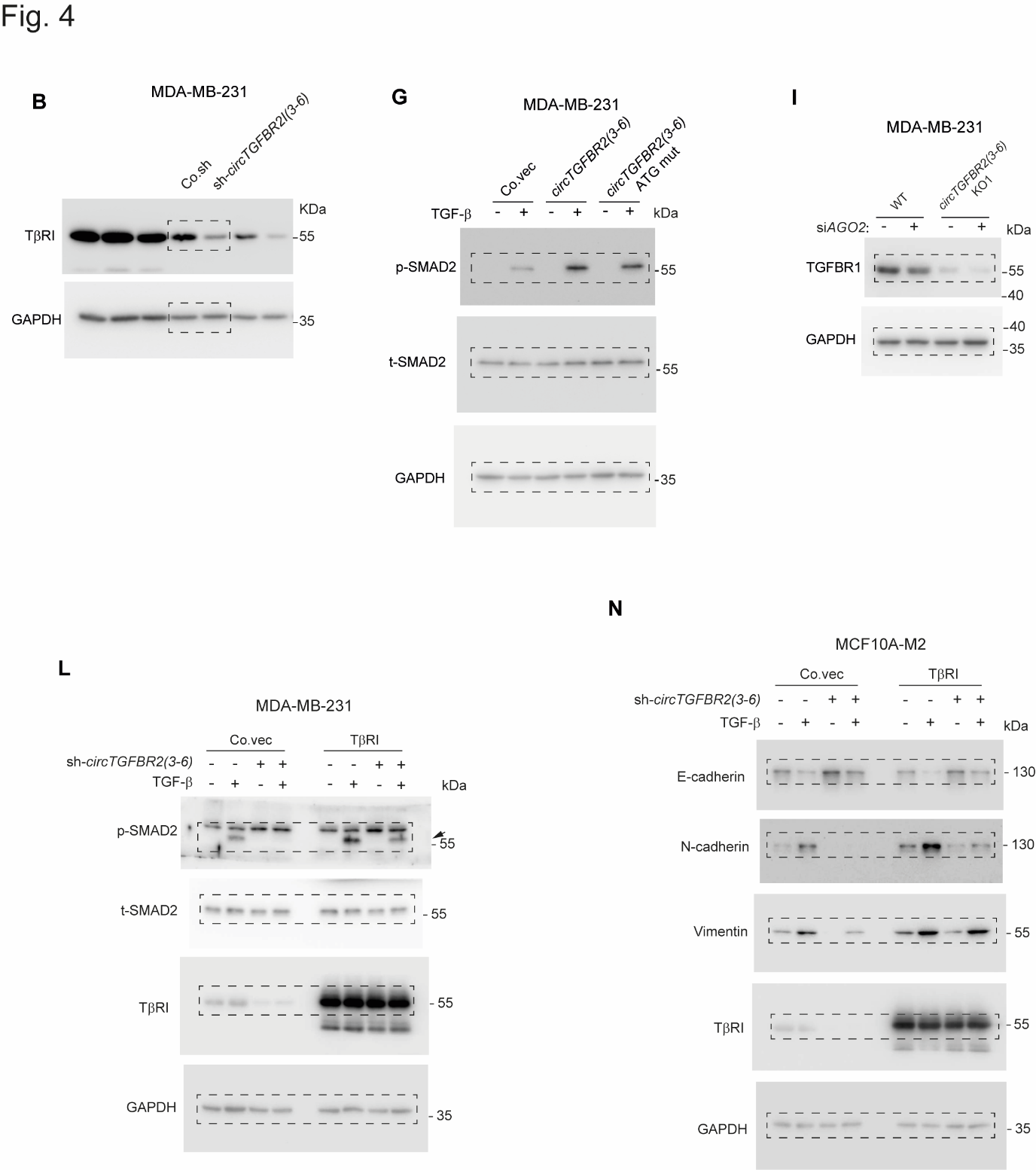

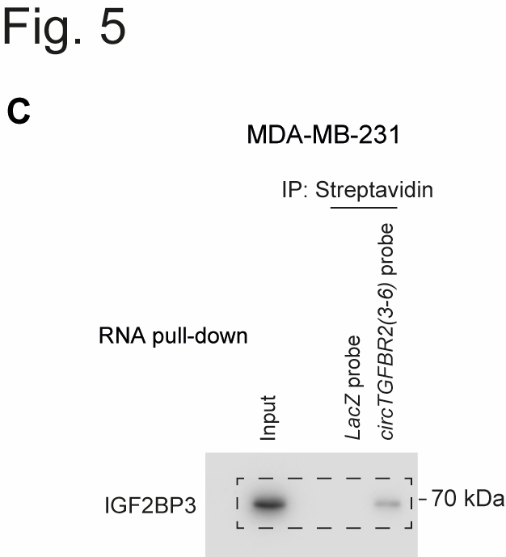

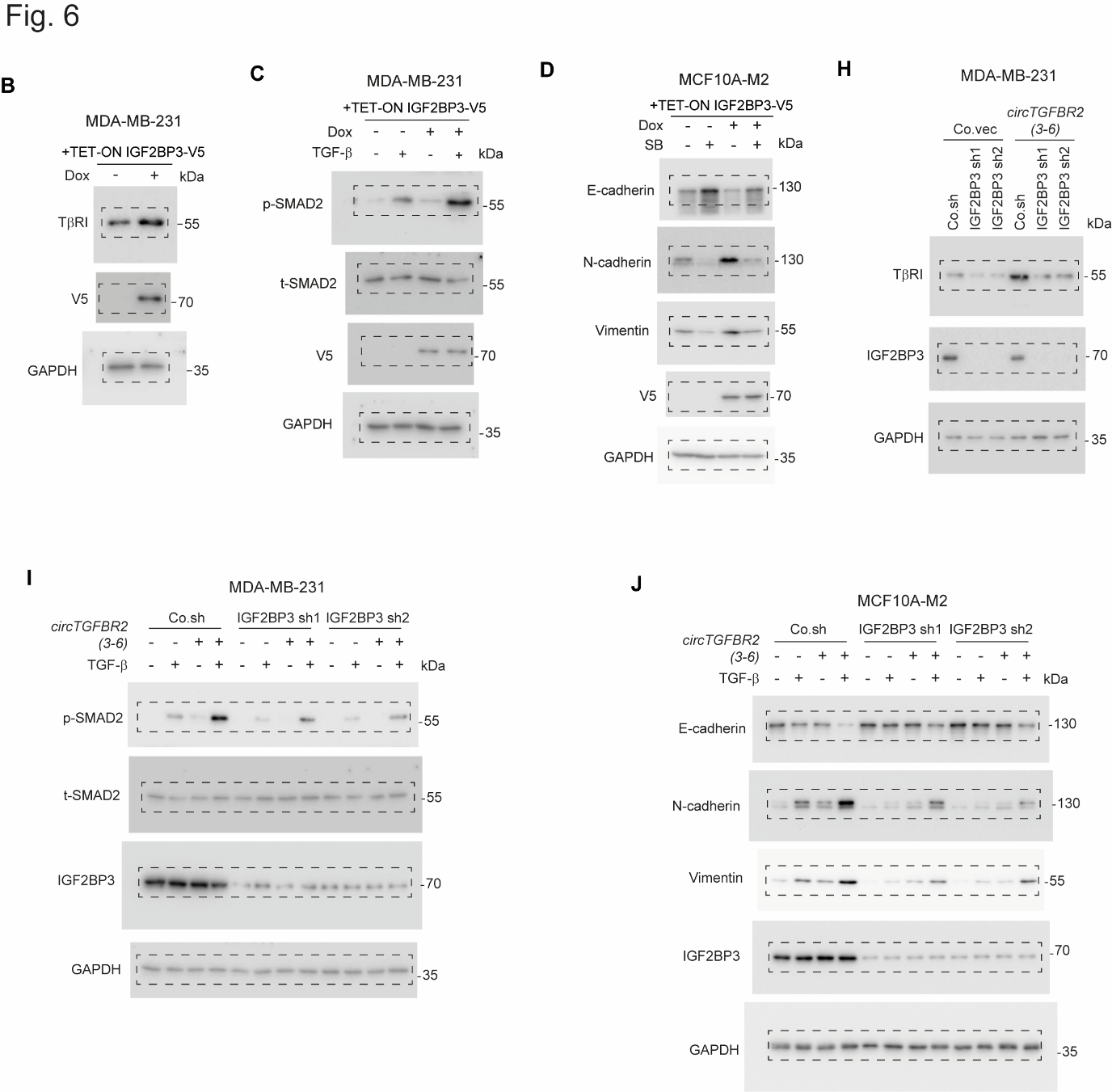

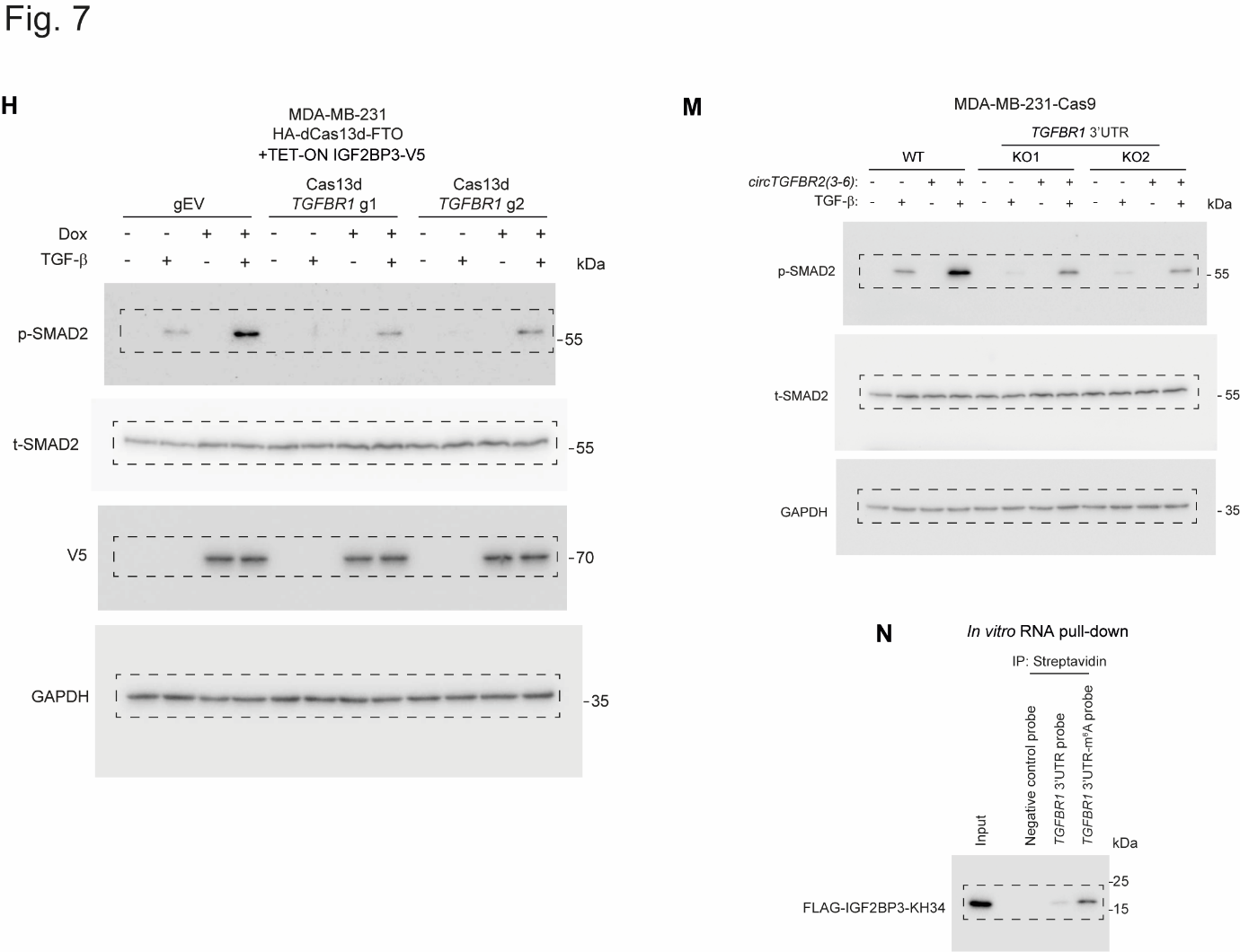


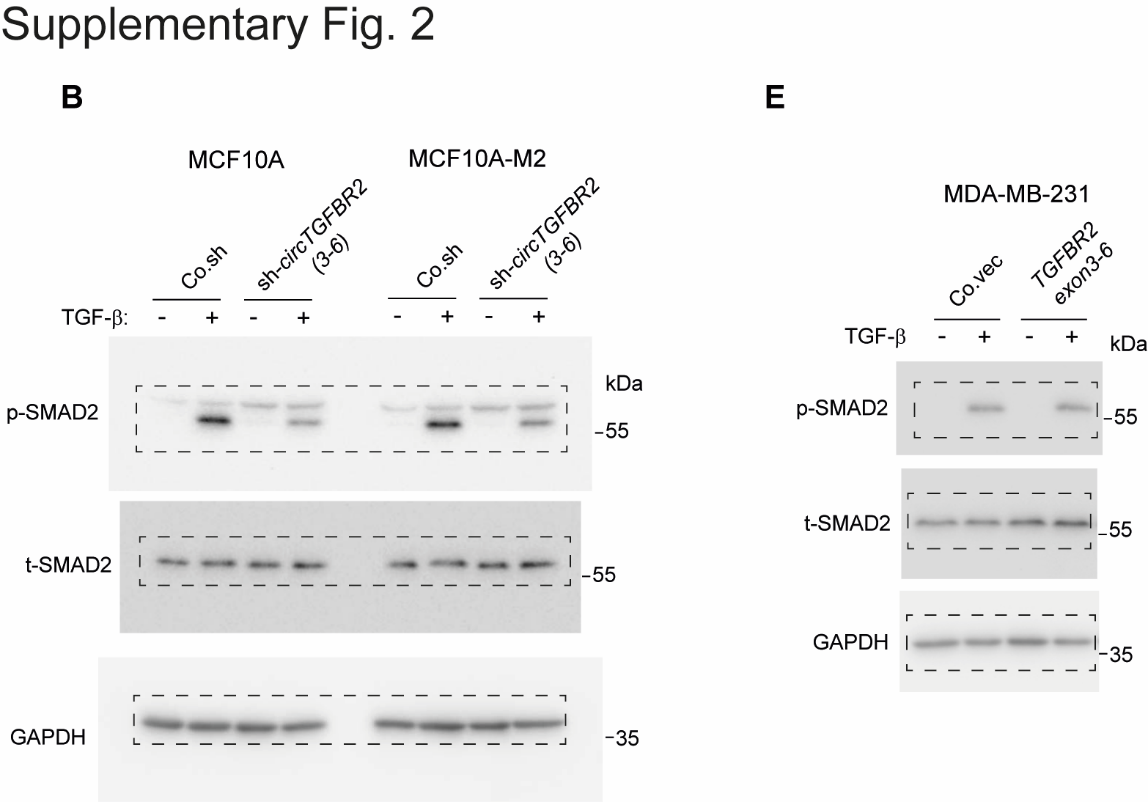


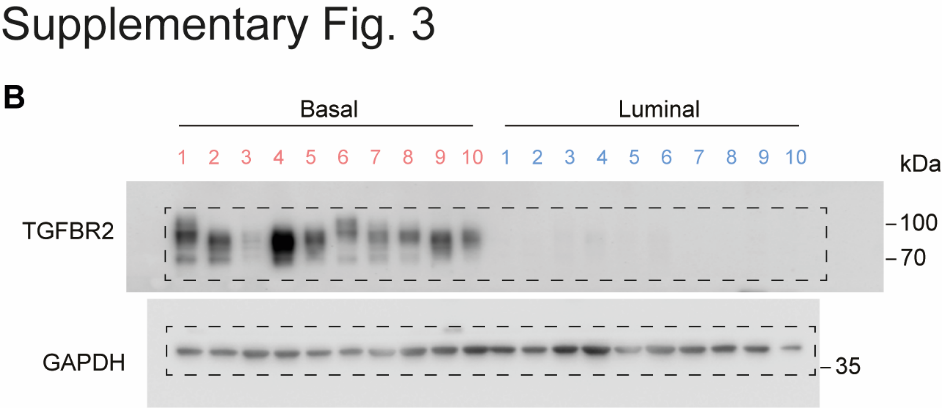


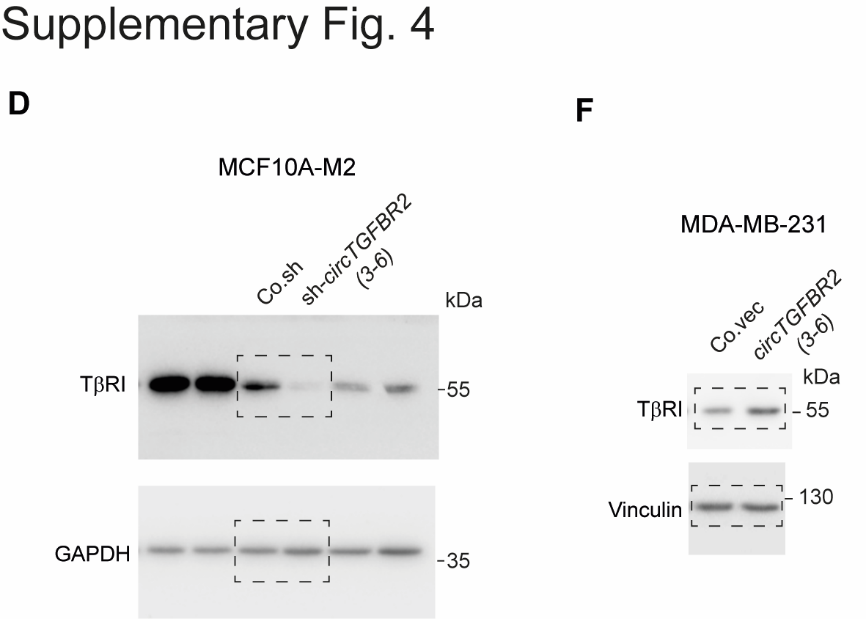


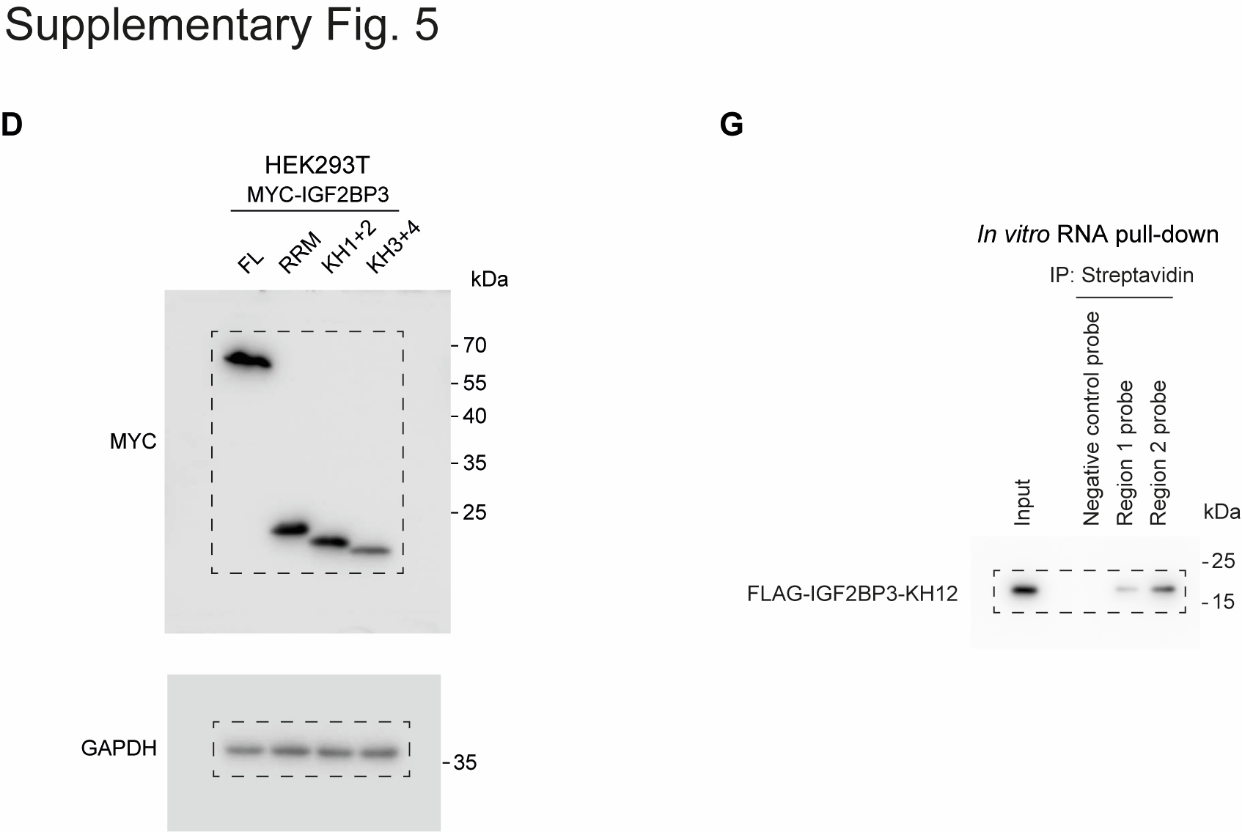


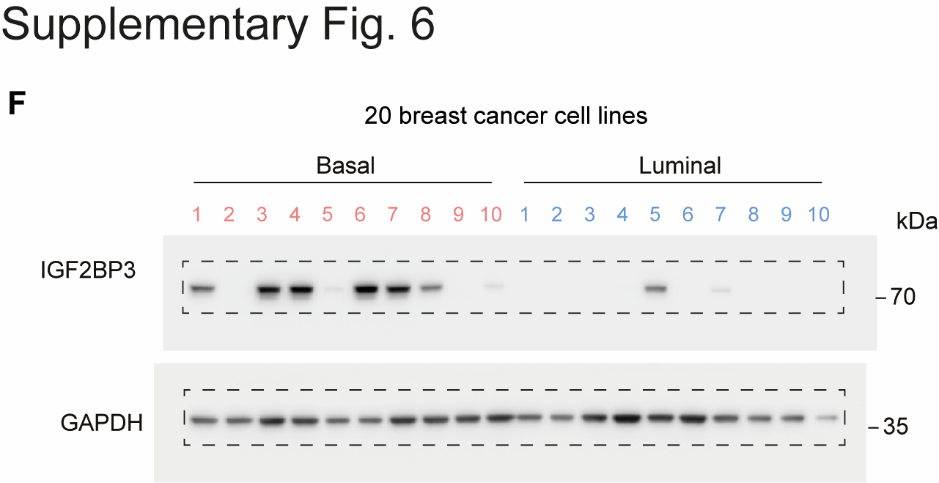


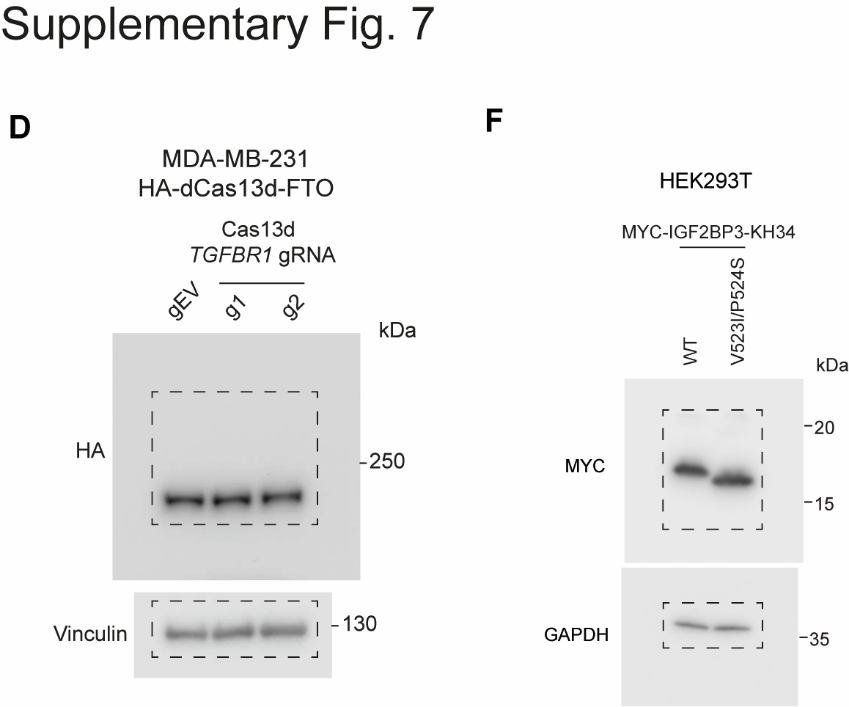

Supplement: Supplementary file 2 — Uncropped blots [file 41418_2025_1597_MOESM2_ESM.docx]
